# DLEU1 promotes oral squamous cell carcinoma progression by activating interferon-stimulated genes

**DOI:** 10.1038/s41598-021-99736-5

**Published:** 2021-10-14

**Authors:** Yui Hatanaka, Takeshi Niinuma, Hiroshi Kitajima, Koyo Nishiyama, Reo Maruyama, Kazuya Ishiguro, Mutsumi Toyota, Eiichiro Yamamoto, Masahiro Kai, Akira Yorozu, Shohei Sekiguchi, Kazuhiro Ogi, Hironari Dehari, Masashi Idogawa, Yasushi Sasaki, Takashi Tokino, Akihiro Miyazaki, Hiromu Suzuki

**Affiliations:** 1 Department of Oral Surgery, Sapporo Medical University School of Medicine, Sapporo, Japan; 2 Department of Molecular Biology, Sapporo Medical University School of Medicine, S1, W17, Chuo-ku, Sapporo, 060-8556 Japan; 3 Project for Cancer Epigenomics, Cancer Institute, Japanese Foundation for Cancer Research, Tokyo, Japan; 4 Department of Gastroenterology and Hepatology, Sapporo Medical University School of Medicine, Sapporo, Japan; 5 Department of Otolaryngology, Sapporo Medical University School of Medicine, Sapporo, Japan; 6 Department of Medical Genome Science, Research Institute for Frontier Medicine, Sapporo Medical University School of Medicine, Sapporo, Japan; 7 Biology Division, Department of Liberal Arts and Sciences, Center for Medical Education, Sapporo Medical University, Sapporo, Japan

**Keywords:** Oral cancer, Head and neck cancer, Long non-coding RNAs, Oncogenes

## Abstract

Long noncoding RNAs (lncRNAs) are deeply involved in cancer development. We previously reported that DLEU1 (deleted in lymphocytic leukemia 1) is one of the lncRNAs overexpressed in oral squamous cell carcinoma (OSCC) cells, where it exhibits oncogenic activity. In the present study, we further clarified the molecular function of DLEU1 in the pathogenesis of OSCC. Chromatin immunoprecipitation-sequencing (ChIP-seq) analysis revealed that DLEU1 knockdown induced significant changes in the levels of histone H3 lysine 4 trimethylation (H3K4me3) and H3K27 acetylation (H3K27ac) in OSCC cells. Notably, DLEU1 knockdown suppressed levels of H3K4me3/ H3K27ac and expression of a number of interferon-stimulated genes (ISGs), including IFIT1, IFI6 and OAS1, while ectopic DLEU1 expression activated these genes. Western blot analysis and reporter assays suggested that DLEU1 upregulates ISGs through activation of JAK-STAT signaling in OSCC cells. Moreover, IFITM1, one of the ISGs induced by DLUE1, was frequently overexpressed in primary OSCC tumors, and its knockdown inhibited OSCC cell proliferation, migration and invasion. These findings suggest that DLEU1 exerts its oncogenic effects, at least in part, through activation of a series ISGs in OSCC cells.

## Introduction

Most oral cancers are classified as oral squamous cell carcinoma (OSCC). According to the latest GLOBOCAN statistics in 2020, there were 377,713 new cases (2.0% of all cancers) reported, leading to 177,757 deaths from disease (1.8% of all cancers) worldwide^1^. The treatment modalities used for oral cancers include surgery, chemotherapy, radiation therapy and immunotherapy, applied alone or in some combination, depending on the location and/or stage of the disease. Although these treatments can be effective, they are often unsatisfactory with advanced or recurrent disease, and the 5-year survival rate among oral cancer patients remains approximately 50%. Molecularly targeted therapies, such as cetuximab (monoclonal antibody for EGFR), are reportedly effective against inoperable and chemotherapy-resistant oral cancers^2,3^. However, molecularly targeted therapies may have specific adverse side-effects, including skin contusions and infusion reactions^2^. In addition, the effectiveness of cetuximab in head and neck squamous cell carcinomas (HNSCCs) is limited due to the multiple mechanisms, including activation of receptor tyrosine kinases and EGFR gene polymorphism, associated with their pathogenesis^4,5^. Consequently, there is still a need to identify novel therapeutic targets in oral cancer.

Genome and transcriptome analyses have revealed that only 2% of the genome is translated into protein, while a large number of non-coding RNAs (ncRNAs) are transcribed from the remaining genomic regions. Moreover, recent studies have demonstrated that ncRNAs, including microRNAs (miRNAs) and long non-coding RNAs (lncRNAs), are deeply involved in the pathogenesis of various disorders, including cancers^6,7^. We previously reported that deleted in lymphocytic leukemia 1 (DLEU1) is a novel OSCC-related lncRNA^8^. DLEU1 is frequently overexpressed in OSCC cell lines, and its knockdown strongly suppresses proliferation, migration, invasion, and in vivo tumor formation by OSCC cells. Levels of DLEU1 expression are elevated in a majority of primary OSCC tumors, and high DLEU1 expression is associated with shorter overall survival in HNSCC patients^8^. Because DLEU1 is located on chromosome 13q14.3, a region frequently deleted in hematopoietic malignancies, DLEU1 was initially identified as a candidate tumor suppressor gene^9,10^. More recently, however, its elevated expression and oncogenicity have been reported in multiple tumor types, including gastric cancer, breast cancer, hepatocellular carcinoma, and renal cell carcinoma^11–14^. Collectively, these results suggest DLEU1 acts as an oncogenic lncRNA in solid tumors and that it could be a potential therapeutic target. In the present study, therefore, we aimed to further clarify the molecular function of DLEU1 and its role in the pathogenesis of OSCC.

## Results

### DLEU1 depletion induces histone modification changes in OSCC cells

**Figure 1:**
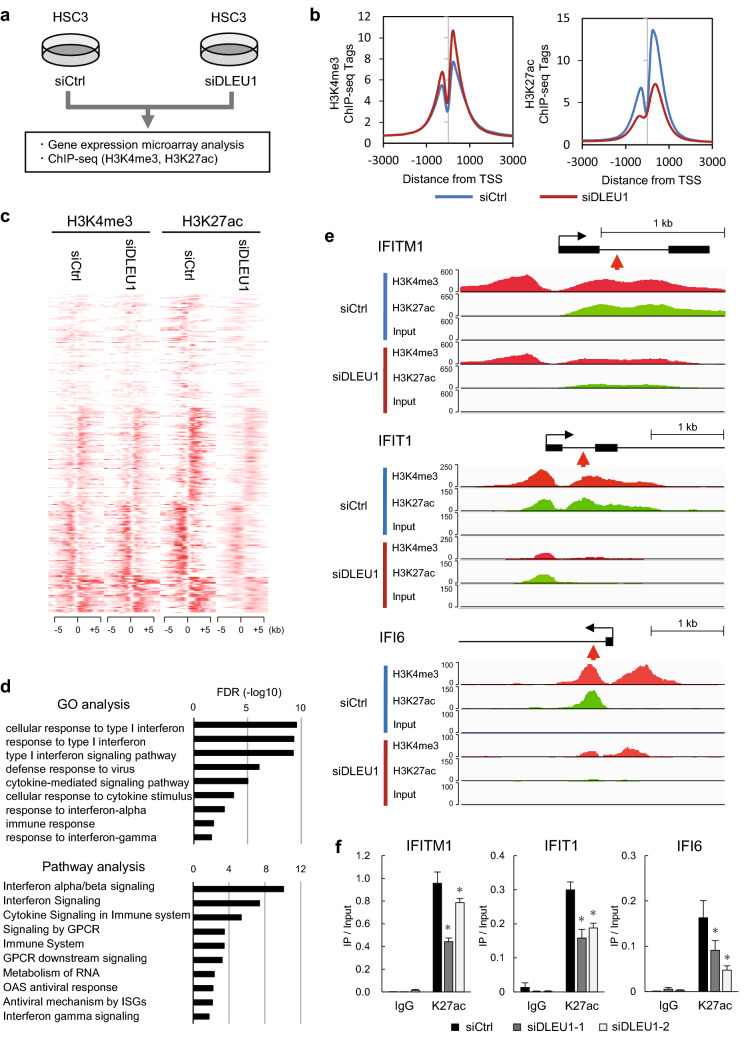
Effects of DLEU1 depletion on histone modification in OSCC cells. (**a**) Workflow of the current study. HSC3 cells were transfected with an siRNA targeting DLEU1 (siDLEU1-2) or a control siRNA, after which microarray and ChIP-seq analyses were performed. (**b**) Metagene plots of H3K4me3 (left) and H3K27ac (right) around the transcription start site (TSS) regions of RefSeq genes in HSC3 cells transfected with the indicated siRNA. (**c**) Heatmaps showing levels of H3K4me3 and H3K27ac around the TSS regions of 300 selected genes. (**d**) Results of Gene Ontology (GO) and pathway analyses of the 300 selected genes. (**e**) ChIP-seq results of representative genes in HSC3 cells transfected with the indicated siRNAs. Gene structures are shown at the top. Red arrows indicate locations analyzed by ChIP-qPCR. (**f**) Results of ChIP-qPCR analysis showing levels of H3K27ac at TSS regions of the indicated genes in HSC3 cells transfected with the indicated siRNA. *P < 0.05.

In a majority of the OSCC cell lines tested, DLEU1 expression was elevated as compared to normal tongue tissue (Supplementary Fig. S1a). To identify downstream targets of DLEU1 in OSCC cells, we first tested the effect of DLEU1 depletion on gene expression and genome-wide histone modification. After confirming that transfecting OSCC cell lines (HSC3, KON and Ca9-22) with siRNA targeting DLEU1 (siDLEU1-1 or siDLEU1-2) depleted its expression (Supplementary Fig. S1b), we performed chromatin immunoprecipitation-sequencing (ChIP-seq) to assess genome-wide histone H3 lysine 4 trimethylation (H3K4me3) and H3K27 acetylation (H3K27ac) in HSC3 cells transfected with siDLEU1-2 or a control siRNA (Fig. 1a). Metagene plot analysis of the transcription start sites (TSSs) of all RefSeq genes revealed that levels of H3K4me3 were moderately elevated while those of H3K27ac were significantly decreased in HSC3 cells, which suggests a number of genes are transcriptionally downregulated by DLEU1 depletion (Fig. 1b). Heatmaps of the 300 selected genes with decreased H3K27ac levels at their TSS regions are shown in Fig. 1c (Supplementary Table S1). Gene ontology and pathway analyses suggested that genes associated with interferon signaling were significantly enriched among the selected genes (Fig. 1d). ChIP-seq results with representative interferon signaling-related genes confirmed the decreased levels of H3K27ac and H3K4me3 at their TSS regions (Fig. 1e, Supplementary Fig. S1c). Downregulation of H3K27ac and H3K4me3 by a second independent siRNA targeting DLEU1 (siDLEU1-1) was then further confirmed with ChIP-quantitative PCR (ChIP-qPCR; Fig. 1f, Supplementary Fig. S1d).

### DLEU1 regulates interferon signaling-related genes in OSCC cells

**Figure 2:**
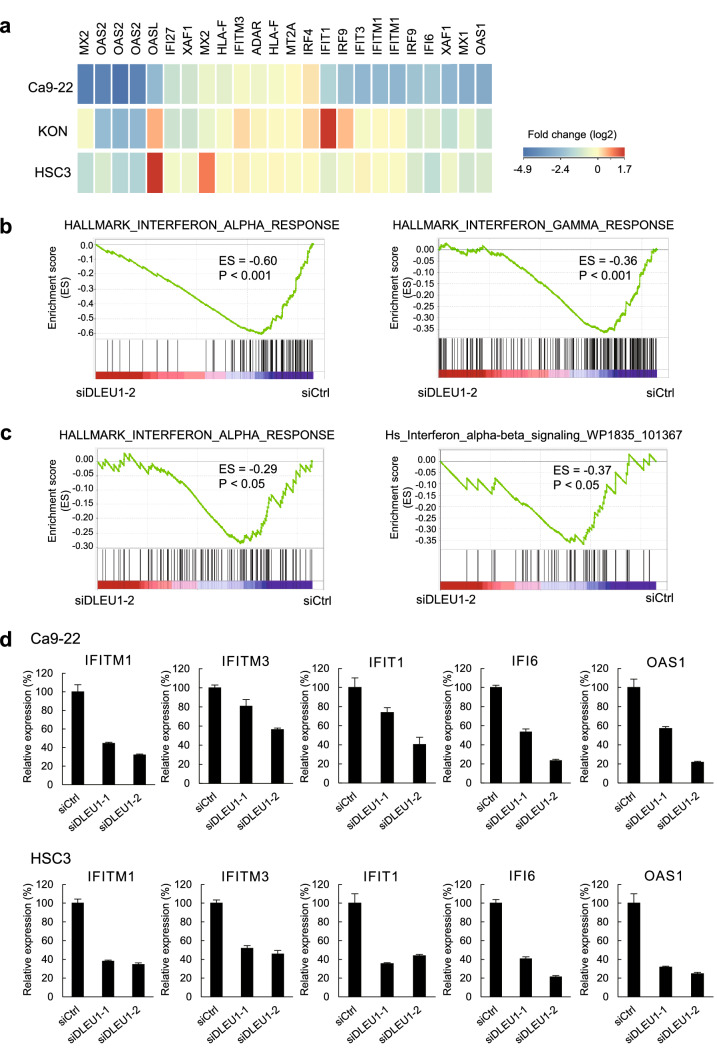
Suppression of interferon signaling-related genes in OSCC cells with DLEU1 knockdown. (**a**) Heatmaps showing microarray results of interferon signaling-related genes in OSCC cell lines with DLEU1 knockdown. Results are normalized to cells transfected with control siRNA. (**b**, **c**) Results of GSEA of the indicated gene sets in Ca9-22 (**b**) and HSC3 (**c**) cells with DLEU1 knockdown. (**d**) Results of qRT-PCR analysis of the indicated ISGs in Ca9-22 (upper) and HSC3 (lower) cells transfected with the indicated siRNA.

We next investigated whether the changes in histone modifications are associated with the expression levels of the genes. Upon reanalyzed the gene expression microarray results obtained from three OSCC cell lines (HSC3, KON and Ca9-22) in our previous study, we found that a series of interferon signaling-related genes were downregulated in cells transfected with siDLEU1-2 (Fig. 2a)^8^. Gene Set Enrichment Analysis (GSEA) confirmed that genes associated with interferon signaling were significantly downregulated by DLEU1 knockdown in OSCC cells (Fig. 2b,c, Supplementary Fig. S2a,b). Suppression of representative interferon-stimulated genes (ISGs) by two independent siRNAs targeting DLEU1 was confirmed by quantitative reverse transcription-PCR (qRT-PCR; Fig. 2d, Supplementary Fig. S2c).

**Figure 3:**
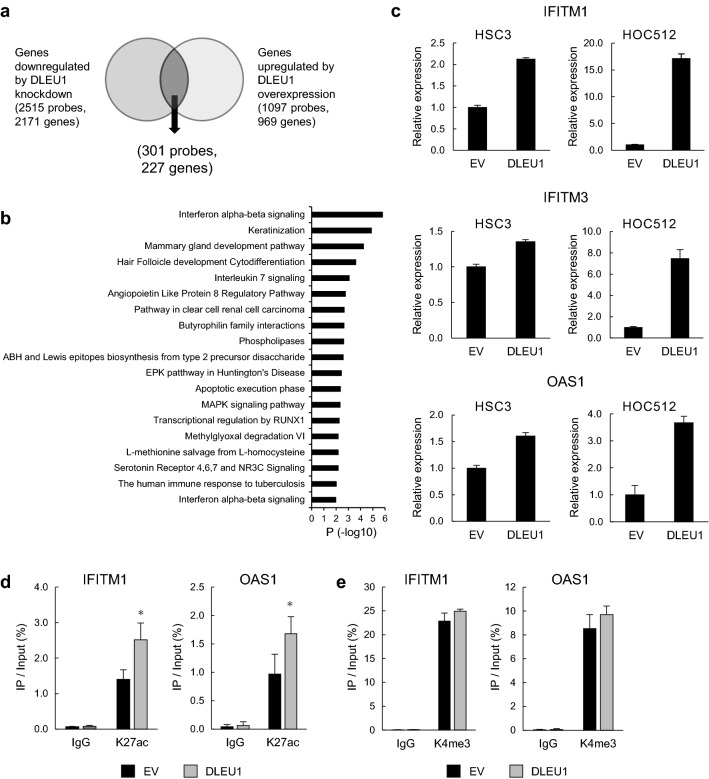
Effects of DLEU1 overexpression on expression and histone modification at ISGs in OSCC cells. (**a**) Venn diagram showing genes downregulated by DLEU1 knockdown and/or upregulated by DLEU1 overexpression detected by microarray analysis of Ca9-22 cells. (**b**) Pathway analysis of selected genes from (**a**). (**c**) qRT-PCR analysis of representative ISGs in the indicated OSCC cells infected with an empty lentivirus vector (EV) or DLEU1 expression vector. (**d**, **e**) ChIP-qPCR analysis showing levels of H3K27ac (**d**) and H3K4me3 (**e**) at TSS regions of the indicated genes in HSC3 cells infected with the indicated lentivirus vectors. *P < 0.05.

To further validate the above findings, we next analyzed the effect of ectopic DLEU1 expression on the gene expression profile in OSCC cells. Microarray analysis of Ca9-22 cells revealed that knocking down DLEU1 downregulated 2515 probe sets (2171 unique genes), while ectopic DLEU1 expression upregulated 1097 probes (969 unique genes), and 301 probes (227 unique genes) overlapped between them (Fig. 3a). Pathway analysis of genes selected from among those affected by DLEU1 suggested that interferon α/β signaling genes were significantly enriched among them (Fig. 3b). Microarray and qRT-PCR analyses confirmed that overexpression of DLEU1 also upregulated ISGs in other OSCC cell lines (Fig. 3c, Supplementary Fig. S3). Moreover, ChIP-qPCR analysis revealed that ectopic DLEU1 expression upregulated levels of H3K27ac at ISGs in OSCC cells, though changes in H3K4me3 levels were marginal (Fig. 3d,e).

### DLEU1 upregulates interferon receptor genes and phosphorylation of STAT1

**Figure 4:**
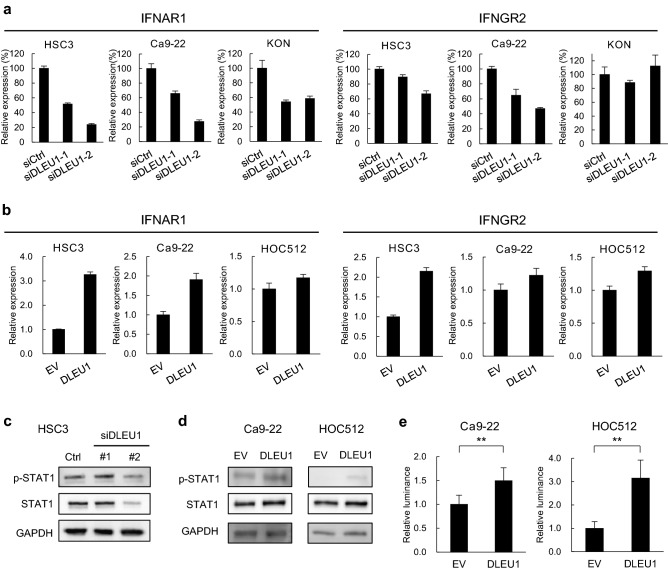
DLEU1 upregulates interferon receptor genes and activates JAK-STAT signaling in OSCC cells. (**a**) qRT-PCR analysis of IFNAR1 (left) and IFNGR2 (right) in OSCC cell lines transfected with the indicated siRNAs. (**b**) qRT-PCR analysis of IFNAR1 (left) and IFNGR2 (right) in OSCC cell lines transfected with the indicated vectors. (**c**, **d**) Western blot analysis of total and phosphorylated STAT1 in the indicated OSCC cells with DLEU1 knockdown (**c**) or ectopic DLEU1 expression (**d**). (**e**) Results of ISRE reporter assays in OSCC cells transfected with the indicated vectors. **P < 0.01.

The results described above suggest that DLEU1 may activate interferon signaling in OSCC cells, though the underlying mechanism remains unclear. Our microarray analysis suggests that several genes encoding interferon receptors were downregulated by DLEU1 knockdown in OSCC cells (Supplementary Fig. S4a). We therefore performed qRT-PCR analysis and found that levels of IFNAR1 and IFNGR2 expression were decreased by DLEU1 knockdown but were increased by ectopic DLEU1 expression in multiple OSCC cell lines (Fig. 4a,b). By contrast, we observed no significant changes in transcription of interferon genes (data not shown). We also observed that levels of phosphorylated STAT1 were decreased in OSCC cells after DLEU1 knockdown (Fig. 4c, Supplementary Fig. S4b). Notably, total STAT1 levels were also suppressed in OSCC cells transfected with siDLEU1-2, which exerted a stronger knockdown effect than siDLEU1-1 (Fig. 4c, Supplementary Fig. S4b). Conversely, levels of phosphorylated STAT1 were elevated in OSCC cells ectopically expressing DLEU1 (Fig. 4d). Luciferase assays using an interferon-stimulated response element (ISRE) reporter confirmed that JAK-STAT signaling was activated by ectopic DLEU1 expression in OSCC cells (Fig. 4e). These results suggest that DLEU1 activates interferon receptor signaling in OSCC cells.

### Functional analysis of DLEU1 and IFITM1 in OSCC cells

**Figure 5:**
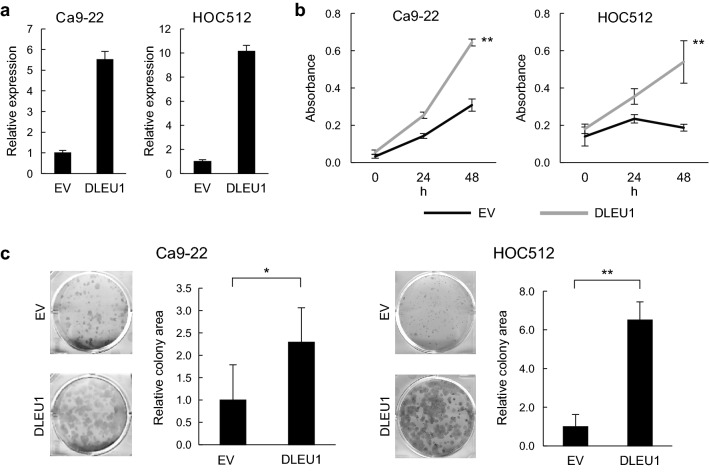
Effects of ectopic DLEU1 expression on OSCC cell proliferation. (**a**) qRT-PCR analysis of DLEU1 in OSCC cells infected with an empty lentivirus vector (EV) or a DLEU1 expression vector. (**b**) Results of cell viability assays in OSCC cells with the indicated lentivirus vectors. (**c**) Results of colony formation assay in OSCC cells infected with indicated lentivirus vectors. *P < 0.05, **P < 0.01.

In an earlier study, we showed that DLEU1 knockdown suppresses OSCC cell proliferation, migration and invasion^8^. To confirm the oncogenic function of DLEU1, we investigated the effect of DLEU1 overexpression on OSCC cell proliferation. After ectopic expression of DLEU1 mediated by a lentiviral vector was confirmed with qRT-PCR (Fig. 5a), we found that it promoted cell viability and colony formation in multiple OSCC cell lines (Fig. 5b,c).

**Figure 6:**
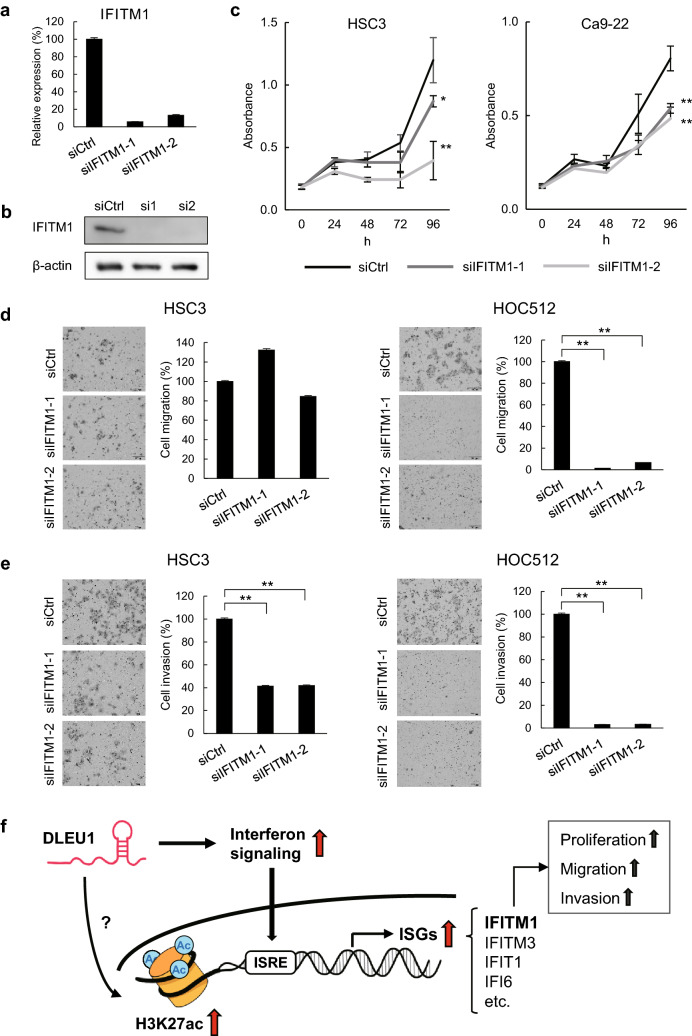
Functional analysis of IFITM1. (**a**) qRT-PCR analysis of IFITM1 in HSC3 cells transfected with the indicated siRNA. (**b**) Western blot analysis of IFITM1 in HSC3 cells transfected with the indicated siRNA. (**c**) Cell viability assays in OSCC cells transfected with the indicated siRNA. (**d**, **e**) Migration (**d**) and invasion (**e**) by OSCC cells transfected with the indicated siRNA. (**f**) A potential role of DLEU1 in OSCC cells. DLEU1 activates interferon signaling and transcription of ISGs, which may promote tumorigenesis. DLEU1 may also affect histone modification at target genes. *P < 0.05, **P < 0.01.

Recent studies have shown that several ISGs are involved in cancer progression^15,16^. Analysis of HNSCC datasets from The Cancer Genome Atlas demonstrated that expression levels of multiple ISGs are higher in primary tumors than normal tissues (Supplementary Fig. S5a). We also found that expression of IFITM1 (interferon-induced transmembrane protein 1), an ISG upregulated by DLEU1 in OSCC cells, is higher in primary OSCC tumors than corresponding normal tissues (Supplementary Fig. S5b,c). Moreover, levels of DLEU1 expression correlated positively with those of IFITM1 in the primary OSCC tissues (Supplementary Fig. S5d). We therefore selected IFITM1 for further functional analysis. Transfecting OSCC cells with an siRNA targeting IFITM1 (siIFITM1-1 or siIFITM1-2) effectively depleted both IFITM1 mRNA and protein (Fig. 6a,b, Supplementary Fig. S5e). IFITM1 knockdown inhibited OSCC cell proliferation (Fig. 6c, Supplementary Fig. S5f.) and suppressed migration by HOC512 cells and invasion by HSC3 and HOC512 cells (Fig. 6d,e). These results suggest that DLEU1 exerts its oncogenic effects, at least in part, through activation of ISGs in OSCC cells (Fig. 6f).

## Discussion

Studies recently showed that DLEU1 is upregulated in various types of cancer and contributes to tumor development and chemoresistance. For instance, DLEU1 is upregulated in colorectal cancer (CRC) and recruits SMARCA1 to epigenetically activate a downstream target gene, KPNA3, which leads to CRC progression^17^. DLEU1 promotes proliferation, migration and invasion by epithelial ovarian cancer cells by interacting with miR-490-3p and altering expression of its target genes, including CDK1^18^. DLEU1 also promotes migration, invasion and EMT by breast cancer cells by targeting miR-300^19^. In bladder cancer, DLUE1 promotes cell proliferation and invasion and confers cisplatin resistance by regulating the miR-99b/HS3ST3B1 axis^20^. And more recently, DLEU1 was shown to promote tumorigenesis in OSCC through regulation of the miR-149/CDK6 axis^21^. Analysis of data from The Cancer Genome Atlas indicated high DLEU1 expression correlated with a poor prognosis in a pan-cancer cohort^22^. Taken together, these reports suggest that DLEU1 promotes tumorigenesis in various organs and that its molecular function varies among tissues.

LncRNAs are involved in a variety of biological processes, including development, cell proliferation, cell cycling, apoptosis and differentiation^23^. LncRNAs exert their effects within physiological and pathological processes acting as various molecular mediators, including guides that recruit target proteins to specific loci, decoys for target proteins or sponges of miRNAs, and scaffolds for creating protein complexes and influencing post-translation modifications^23^. Several lncRNAs are known to act as epigenetic regulators and to promote tumorigenesis by altering histone modifications at their target genes. For instance, nuclear paraspeckle assembly transcript 1 (NEAT1) directly interacts with BRG1 and silences GADD45A gene via modulating H3K27me3 and H3K4me3 in the promoter region^24^. CDKN2B-AS1 recruits CREB-binding protein (CBP) and SET and MYND domain-containing 3 (SMYD3) and activates the target gene NUF2 by upregulating H3K27ac and H3K4me3^25^. One recent study showed that upregulation DLEU1 is associated with increased levels of H3K4me3 and H3K27ac at its promoter region in multiple cancer cell lines, and DLEU1 also upregulates its neighboring gene SRP4 (KPNA3) by increasing H3K27ac at the SRP4 locus^22^. In the present study, we found that DLEU1 depletion alters levels of H3K4me3 and H3K27ac in the TSS regions of a large number of genes in OSCC cells. Notably, a series of ISGs were significantly enriched among the affected genes.

ISGs are induced by several types of interferons, and it is well known that interferons exhibit antitumor activities via direct effects on cancer cells or activation of immune cells mediating antitumor immunity^26^. Secreted interferons bind to cell surface interferon receptors and activate the JAK-STAT signaling cascade. STAT family proteins phosphorylated and activated by JAK form homo- or heterodimers, localize to the nucleus, bind to specific elements in the promoter region of a series of ISGs, and upregulate their transcription^27^. Recent studies revealed that several ISGs exert oncogenic effects that are independent of innate immunity. For instance, interferon alpha-inducible protein 6 (IFI6) is reportedly necessary for oncogenic NRAS-induced transformation and melanoma growth^15^. KLF7 promotes pancreatic cancer growth and metastasis by upregulating ISGs that include IFI6, interferon-induced protein with tetratricopeptide repeats 1 (IFIT1) and IFIT3^28^. IFITM3 promotes bone metastasis in prostate cancer by activating TGF-β signaling^29^. In addition, IFITM3 was recently shown to promote malignant transformation of B cells by amplifying PI3K signaling^30^. Potential oncogenic functions of ISGs were also observed in OSCC. IFIT1 and IFIT3 reportedly promote OSCC metastasis, though their overexpression enhances the antitumor effect of gefitinib by increasing levels of phosphorylated EGFR^16^. In the present study, we found that IFITM1, which is one of the ISGs upregulated by DLEU1, contributes to OSCC cell proliferation, migration and invasion.

IFITM1 expression is stimulated by interferons and plays a role in antiviral immune responses^31,32^. However, recent studies have shown that IFITM1 is also associated with the development and progression of multiple types of malignancies and that it is a potential prognostic marker and therapeutic target. For instance, overexpression of IFITM1 has been observed in colorectal, ovarian and esophageal cancers^33–35^, and is associated with poor differentiation and poorer outcomes in CRC^36^. IFITM1 is also reportedly one of the ISGs associated with resistance to chemotherapy and radiotherapy in breast cancer^37^. Consistent with those observations, a recent study showed that IFITM1 is a radioresistance-related gene in oral cancer and that a combination of IFITM1 knockdown and radiotherapy effectively inhibited survival of OSCC cells^38^. Within the context of those reports, our findings suggest that the oncogenic function of DLEU1 is mediated, at least in part, through activation of a series of ISGs, including IFITM1.

There are several limitations to this study. First, we found that depletion of DLEU1 led to significant downregulation of H3K27ac in a large number of TSS regions in OSCC cells, but the underlying mechanism is not fully understood. Downregulation of interferon signaling may be responsible for the decreased H3K4me3/H3K27ac levels in ISGs, but the mechanism to downregulate genes unrelated to interferon signaling remains unclear. It is also possible that DLEU1 directly alters histone modification at target genes through interaction with epigenetic modifiers. Second, the mechanism by which DLEU1 activates interferon signaling in OSCC cells is not fully elucidated. Upregulation of interferon receptor genes may be one of the mechanisms, but DLEU1 may also activate JAK-STAT1 signaling via unknown molecular activity. Further studies, including identification of molecules that interact with DLEU1 in OSCC cells, will be required to address these issues. Third, it remains unclear whether DLEU1 inhibition increases the efficacy of other treatments, including chemotherapy and molecularly targeted therapies. Further studies, including in vivo experiments, will be necessary to clarify the oncogenic effect of DLEU1 and its usefulness as a therapeutic target in OSCC.

In summary, we found that DLEU1 activates a number of interferon signaling-related genes in OSCC cells. Genes upregulated by DLEU1 include multiple ISGs whose oncogenic functions have been reported in human malignancies (e.g., IFITM1). Although recent studies revealed the elevated expression and tumor promoting functions of DLEU1 in various solid tumors, the present study is the first to show that DLEU1 exerts its effects, at least in part, through activation of ISGs. These results suggest DLEU1 is a potential therapeutic target in OSCC, and further studies to clarify its clinical usefulness are warranted.

## Materials and methods

### Cell lines and tissue samples

OSCC cell lines, HSC3, Ca9-22, KON (obtained from the Japanese Collection of Research Bioresources, Tokyo, Japan) and HOC521 (kindly provided by Dr. N. Kamata, Tokushima University, Tokushima, Japan) were cultured as described previously^8,39^. A series of 32 samples of primary OSCC tissue and 17 samples of adjacent nontumorous tissue were obtained as described^8^. Total RNA was extracted using an RNeasy Mini kit (Qiagen, Hilden, Germany) according to the manufacturer’s instructions.

### Reverse transcription-PCR

Single-stranded cDNA was prepared using PrimeScript RT Master Mix Perfect Realtime (TaKaRa Bio, Kusatsu, Japan). qRT-PCR was performed using PowerUp SYBR Green Master Mix (Invitrogen by Thermo Fisher Scientific) with a 7500 Fast Real-Time PCR System (Thermo Fisher Scientific). Relative expression levels of the target gene were determined using an endogenous housekeeping gene beta-2-microglobulin (B2M) as an internal control. All primers were designed using Primer3 (https://bioinfo.ut.ee/primer3-0.4.0/). The sequences of the primers are listed in Supplementary Table S2.

### siRNA and expression vectors

Predesigned and custom-designed siRNAs were purchased from Sigma-Aldrich (St Louis, MO, USA). siRNAs (20 µM) were transfected into OSCC cell lines using Lipofectamine RNAiMax (Thermo Fisher Scientific, Waltham, MA, USA) according to manufacturer’s instructions. The siRNA sequences are listed in Supplementary Table S3. The full-length DLEU1 cDNA was amplified by PCR using cDNA derived from KON cells as a template and then cloned into pLenti6/V5‐DEST. Lentiviral expression vectors (lenti-DLEU1 and an empty vector) were constructed using a ViraPower Lentiviral Expression System (Thermo Fisher Scientific). OSCC cells were infected with lentivirus vectors and selected for 2 weeks using 6 mg/ml blasticidin (Thermo Fisher Scientific).

### Chromatin immunoprecipitation (ChIP)

Chromatin immunoprecipitation (ChIP) was performed as described previously^40^. Cells were transfected with siRNAs as described above and incubated for 72 h. Aliquots of cells (1 × 10^6^) were then fixed and crosslinked, after which samples were immunoprecipitated for 12 h at 4 °C using a rabbit anti-acetylated histone H3 lysine 27 (H3K27ac) pAb (#39,133; Active Motif, Carlsbad, CA, USA), a rabbit anti-trimethylated histone H3 lysine 4 (H3K4me3) mAb (#04–745; Merck, Darmstadt, Germany), or normal mouse IgG (#140–09,511, Fujifilm, Tokyo, Japan), which served as a control. For ChIP-seq analysis, samples were sequenced using an Ion Proton System (Thermo Fisher Scientific). Sequencing data were mapped to the human genome hg19 using bowtie2, after which peaks were called using MACS2 and were annotated using HOMER v4.11. ChIP-seq results were visualized using Microsoft Excel, Java TreeView and Integrative Genomics Viewer. ChIP-qPCR was carried out using PowerUp SYBR Green Master Mix (Thermo Fisher Scientific). The sequences of the PCR primers are listed in Supplementary Table S2. The Gene Expression Omnibus accession number for the microarray data is GSE178613.

### Gene expression microarray analysis

OSCC cells were infected with lentivirus vectors as described above. Gene expression microarray analysis was carried out using SurePrint G3 Human GE microarray v3 (Agilent Technologies) as described previously^41^. The microarray data were analyzed using GeneSpring GX version 13 (Agilent Technologies). The Gene Expression Omnibus accession number for the microarray data is GSE178613.

### Cell viability assays

OSCC cells (5 × 10^3^ cells/well in 96 well plates) were transfected with siRNAs or infected with lentiviral vectors as described above. Cell viability assays were carried out using a Cell Counting kit-8 (Dojindo, Kumamoto, Japan) according to the manufacturer’s instructions.

### Cell migration and invasion assays

Cell migration and invasion assays were performed as described previously^8^. Briefly, OSCC cells were transfected with siRNAs as described above and incubated for 48 h. Transwell chambers were used for cell migration (BioCoat Control Insert 24-well plate 8.0 μm; Corning Inc., Corning, NY, USA) and invasion (BioCoat Matrigel Invasion Chamber 24-well plate 8.0 μm; Corning Inc.) analyses. The numbers of migrated or invaded cells were determined microscopically by counting in eight randomly selected microscopic fields per membrane.

### Luciferase assays

To establish an ISRE reporter (pNL3.2-ISRE), a pNL3.2-NF-κB-RE vector (Promega, Madison, WI, USA) was digested with SacI and BglII to remove the NF-κB-responsive element, after which a DNA fragment containing ISRE was cloned into the vector. OSCC cells were infected with lenti-DLEU1 or empty vector as described above. Cells (1 × 10^5^ cells/well in 96-well plates) were then transfected with 12.5 ng of pGL4.53 (Promega) and 37.5 ng of pNL3.2-ISRE using Lipofectamine 3000 (Thermo Fisher Scientific, Waltham, MA, USA) according to the manufacturer's instructions. Luciferase activities were measured 24 h after transfection using a Nano-Glo Dual-Luciferase Reporter assay system (Promega).

### Western blot analysis

Western blot analysis was performed as described previously^42^. A mouse monoclonal anti-β-actin mAb (1:5000 dilution, #A5441; Sigma-Aldrich, St. Louis, MO, USA), a mouse monoclonal anti-GAPDH mAb (1:5000 dilution, Proteintech, Rosemont, IL, USA), a rabbit anti‐STAT mAb (1:1000 dilution, Cell signaling Technology, Danvers, MA, USA), a rabbit anti-pSTAT mAb (1:1000 dilution, Cell signaling Technology), and a rabbit anti-IFITM1 pAb (1:1000 dilution, Cell signaling Technology) were used. Signals were detected using HRP‐conjugated secondary antibodies (Cell Signaling Technology). Luminescent signals were detected using an ImageQuant LAS‐4000 mini image reader (GE Healthcare Japan, Tokyo, Japan).

#### Data analysis

RNA sequencing (RNA-seq) data from HNSCC in The Cancer Genome Atlas datasets were obtained from UCSC Xena (http://xena.ucsc.edu/). Quantitative variables were analyzed using Student’s t test. Values of *P* < 0.05 (2 sided) were considered statistically significant. Statistical analyses were carried out using EZR version 1.32 (Saitama Medical Center, Jichi Medical University, Saitama, Japan).

## Supplementary Information

Supplementary Information.
