# Supplementary material for: DLEU1 promotes oral squamous cell carcinoma progression by activating interferon-stimulated genes
Source: Sci Rep. 2021 Oct 14;11:20438. doi: 10.1038/s41598-021-99736-5 (PMC8516910; doi:10.1038/s41598-021-99736-5)
Supplement: Supplementary file 1 — Supplementary Information. [file 41598_2021_99736_MOESM1_ESM.pdf]

Supplementary information

DLEU1 promotes oral squamous cell carcinoma progression by activating interferon-stimulated genes

Yui Hatanaka[1,2], Takeshi Niinuma[2], Hiroshi Kitajima[2], Koyo Nishiyama[1], Reo Maruyama[3], Kazuya Ishiguro[2,4], Mutsumi Toyota[2], Eiichiro Yamamoto[2], Masahiro Kai[2], Akira Yorozu[2,5], Shohei Sekiguchi[1,2], Kazuhiro Ogi[1], Hironari Dehari[1], Masashi Idogawa[6], Yasushi Sasaki[7], Takashi Tokino[6], Akihiro Miyazaki[1], Hiromu Suzuki[2]

[1]Department of Oral Surgery, Sapporo Medical University School of Medicine, Sapporo, Japan
[2]Department of Molecular Biology, Sapporo Medical University School of Medicine, Sapporo, Japan
[3]Project for Cancer Epigenomics, Cancer Institute, Japanese Foundation for Cancer Research, Tokyo, Japan
[4]Department of Gastroenterology and Hepatology, Sapporo Medical University School of Medicine, Sapporo, Japan
[5]Department of Otolaryngology, Sapporo Medical University School of Medicine, Sapporo, Japan
[6]Department of Medical Genome Science, Research Institute for Frontier Medicine, Sapporo Medical University School of Medicine, Sapporo, Japan
[7]Biology Division, Department of Liberal Arts and Sciences, Center for Medical Education, Sapporo Medical University, Sapporo, Japan.

Supplementary Figure S1

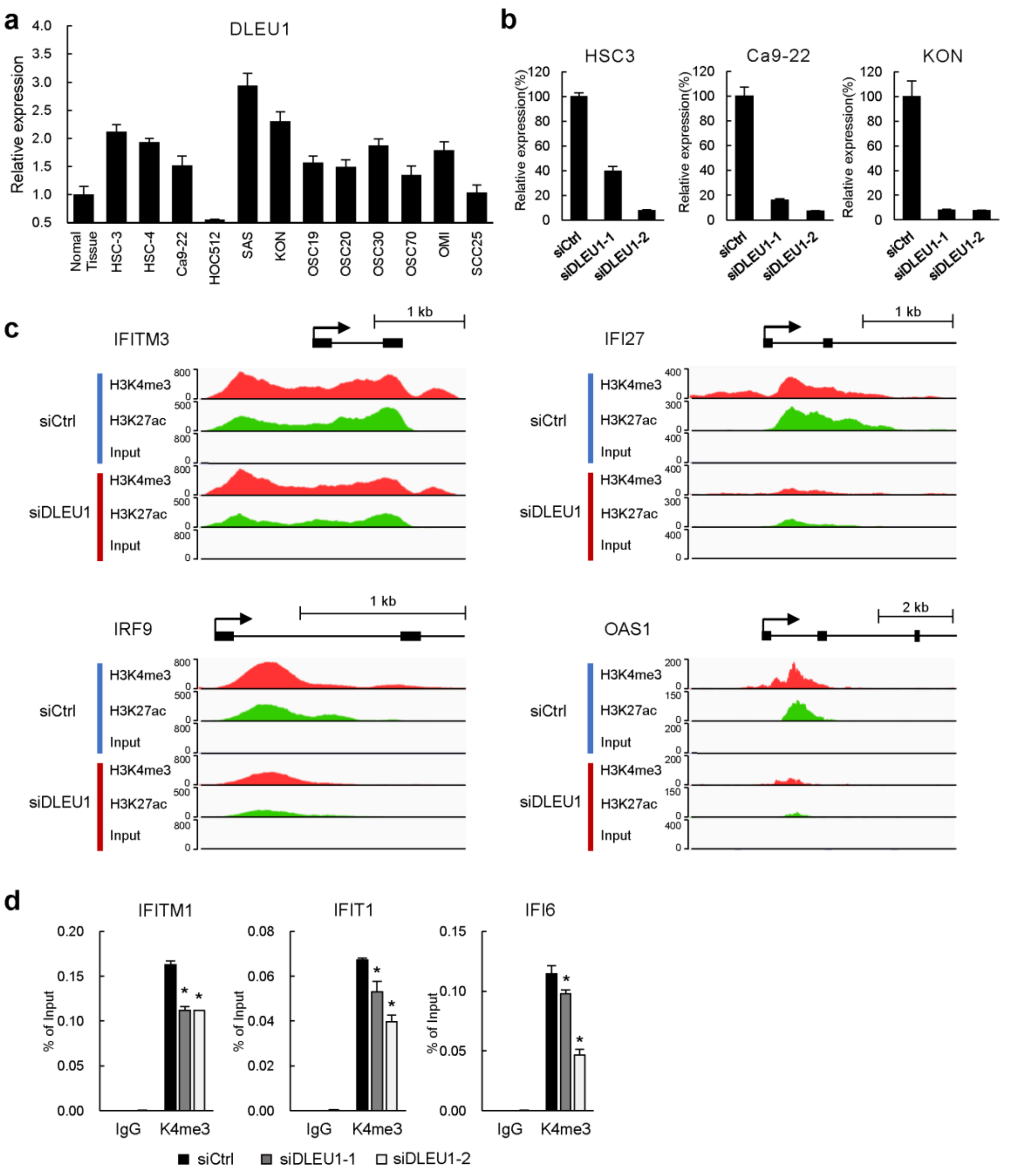

Supplementary Figure S1

Effects of DLEU1 depletion on histone modification at interferon-signaling related genes in OSCC cells. (a) qRT-PCR analysis of DLEU1 in a series of OSCC cell lines and in normal tongue tissue. (b)

qRT-PCR analysis of DLEU1 in the indicated OSCC cell lines transfected with control siRNA (siCtrl) or siRNA targeting DLEU1 (siDLEU1-1 or siDLEU1-2). (c) Results of ChIP-seq showing levels of H3K4me3 and H3K27ac at TSS regions of representative interferon-stimulated genes (ISGs) in HSC3 cells transfected with the indicated siRNAs. Gene structures are shown at the top. (d) Results of ChIP-qPCR analysis showing levels of H3K4me3 at the TSS regions of representative ISGs in HSC3 cells transfected with the indicated siRNA. *$P < 0.05$.

Software used to draw Figure S1c

Integrative Genomics Viewer 2.6 https://software.broadinstitute.org/software/igv/

Supplementary Figure S2

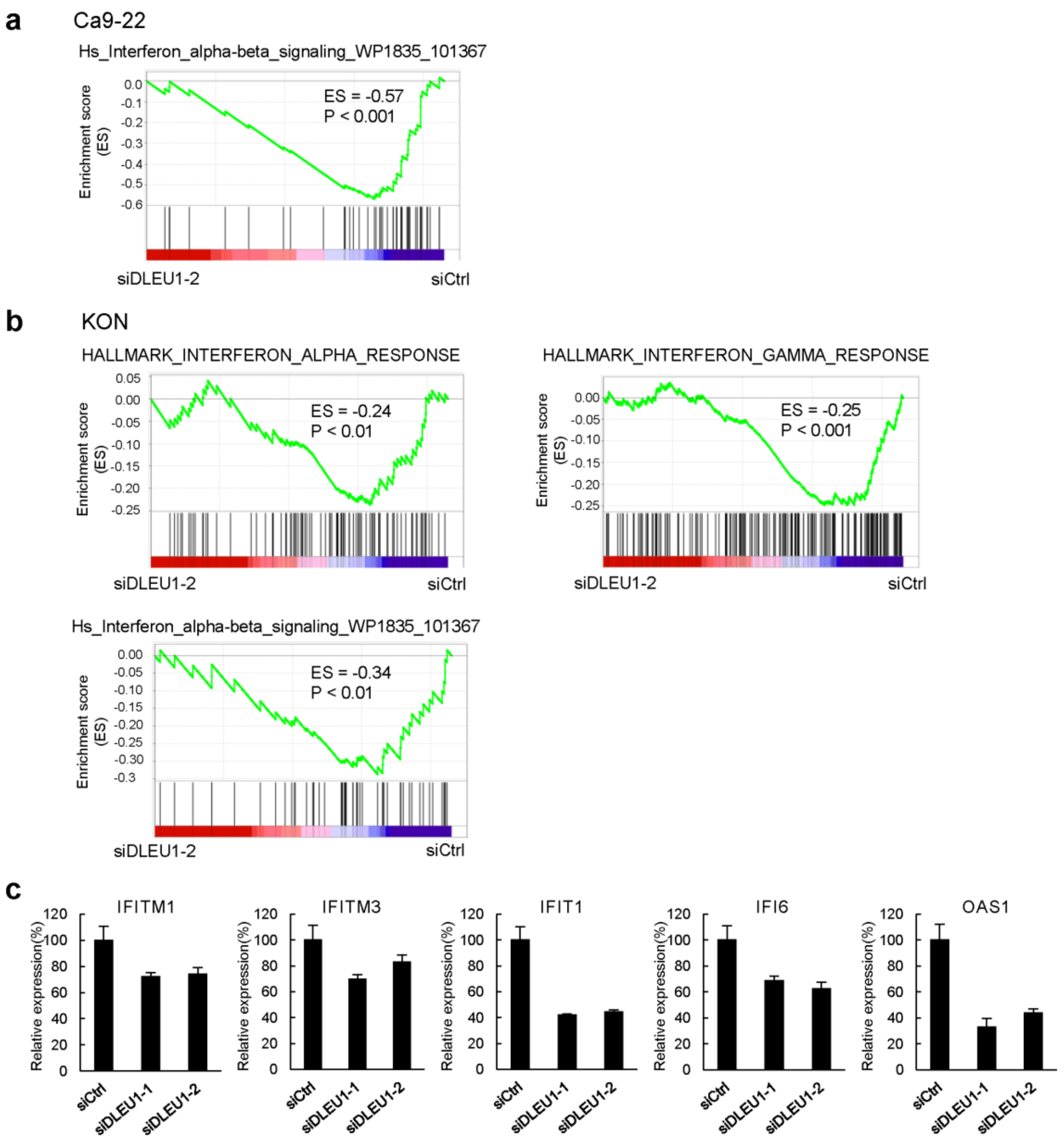

Supplementary Figure S2

DLEU1 knockdown suppresses interferon signaling-related genes in OSCC cells. (a) Gene Set Enrichment Analysis (GSEA) of interferon α/β signaling genes in Ca9-22 cells. (b) GSEA of the indicated gene sets in KON cells. (c) qRT-PCR analysis of the indicated ISGs in KON cells transfected with the indicated siRNA.

Supplementary Figure S3

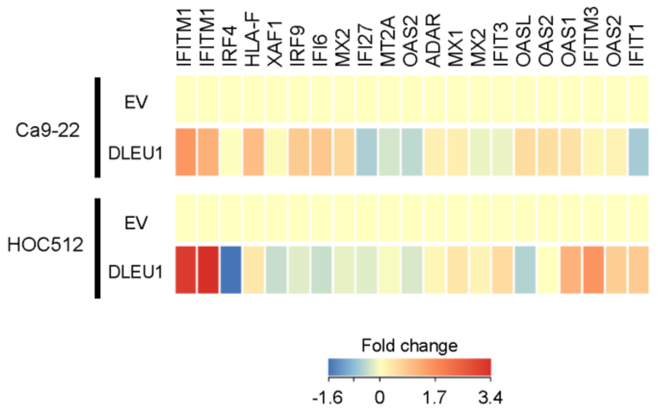

Supplementary Figure S3

DLEU1 overexpression upregulates interferon-signaling related genes in OSCC cells. Heatmaps showing microarray results for the indicated genes in Ca9-22 (upper) and HOC512 (lower) cells infected with an empty lentivirus vector (EV) or a DLEU1 expression vector. Results are normalized to cells with an empty vector.

Software used to draw Figure S3

GeneSpring GX version 13 https://www.agilent.com/en/product/software-informatics/genomics-software-informatics/gene-expression/genespring-gx

Supplementary Figure S4

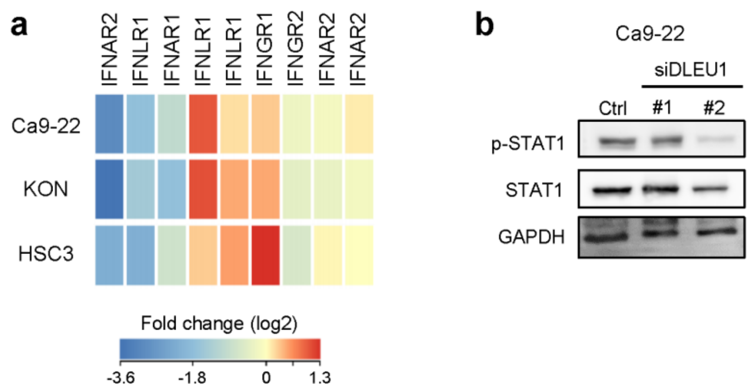

Supplementary Figure S4

DLEU1 upregulates interferon receptor genes and activates JAK-STAT signaling in OSCC cells. (a) Heatmaps showing microarray results for interferon receptor genes in the indicated OSCC cell lines transfected with siRNA targeting DLEU1. Results are normalized to cells transfected with control siRNA. (b) Western blot analysis of total and phosphorylated STAT1 in Ca9-22 cells transfected with the indicated siRNA.

Software used to draw Figure S4a

GeneSpring GX version 13 https://www.agilent.com/en/product/software-informatics/genomics-software-informatics/gene-expression/genespring-gx

Supplementary Figure S5

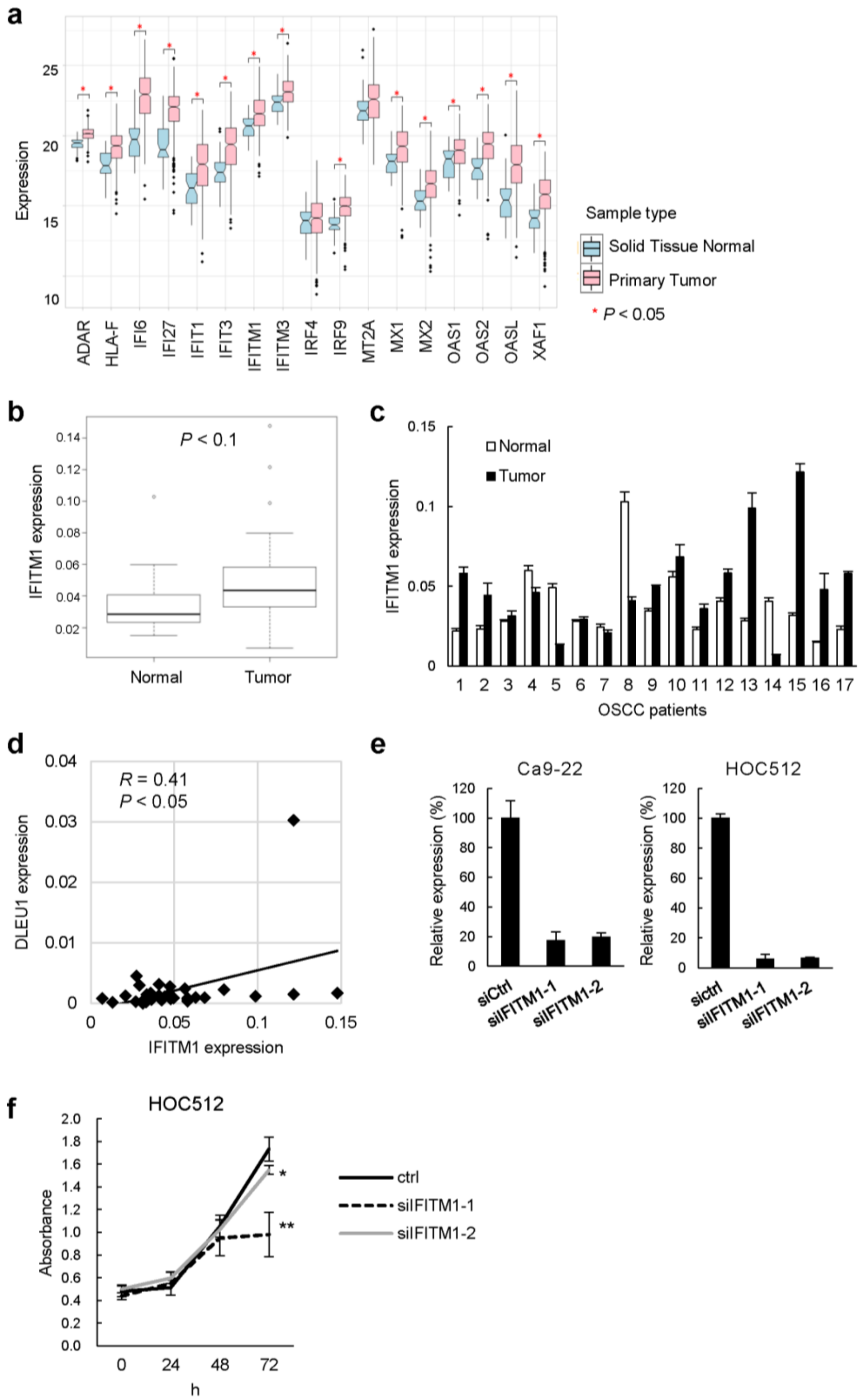

Supplementary Figure S5

Expression and functional analysis of interferon-stimulated genes (ISGs) in OSCC. (a) Summarized RNA-seq results from datasets for the indicated ISGs in head and neck squamous cell carcinoma (HNSCC) primary tumor and normal tissues in The Cancer Genome Atlas. (b) Summarized qRT-PCR results for IFITM1 in primary OSCC tumors (n = 32) and adjacent normal tissues (n = 17). (c) qRT-PCR analysis of IFITM1 in paired normal tissues and primary OSCC tumors. (d) Correlation between expression levels of DLEU1 and those of IFITM1 in primary OSCC tumors (n = 32). Pearson's correlation coefficient (*R*) is shown. (e) qRT-PCR analysis of IFITM1 in indicated OSCC cell lines transfected with the indicated siRNA. (f) Cell viability assays in HOC512 cells transfected with the indicated siRNA. *$P < 0.05$, **$P < 0.01$.

Hatanaka *et al*. Original images of western blots

Figure 4c

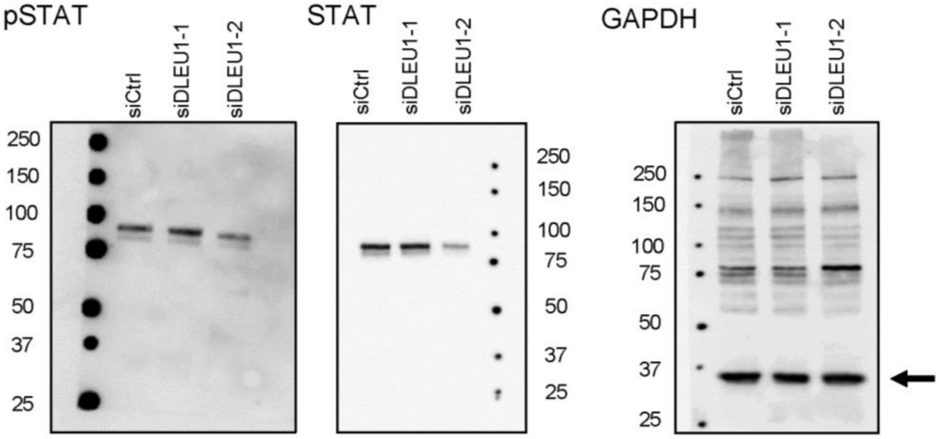

Figure 4d

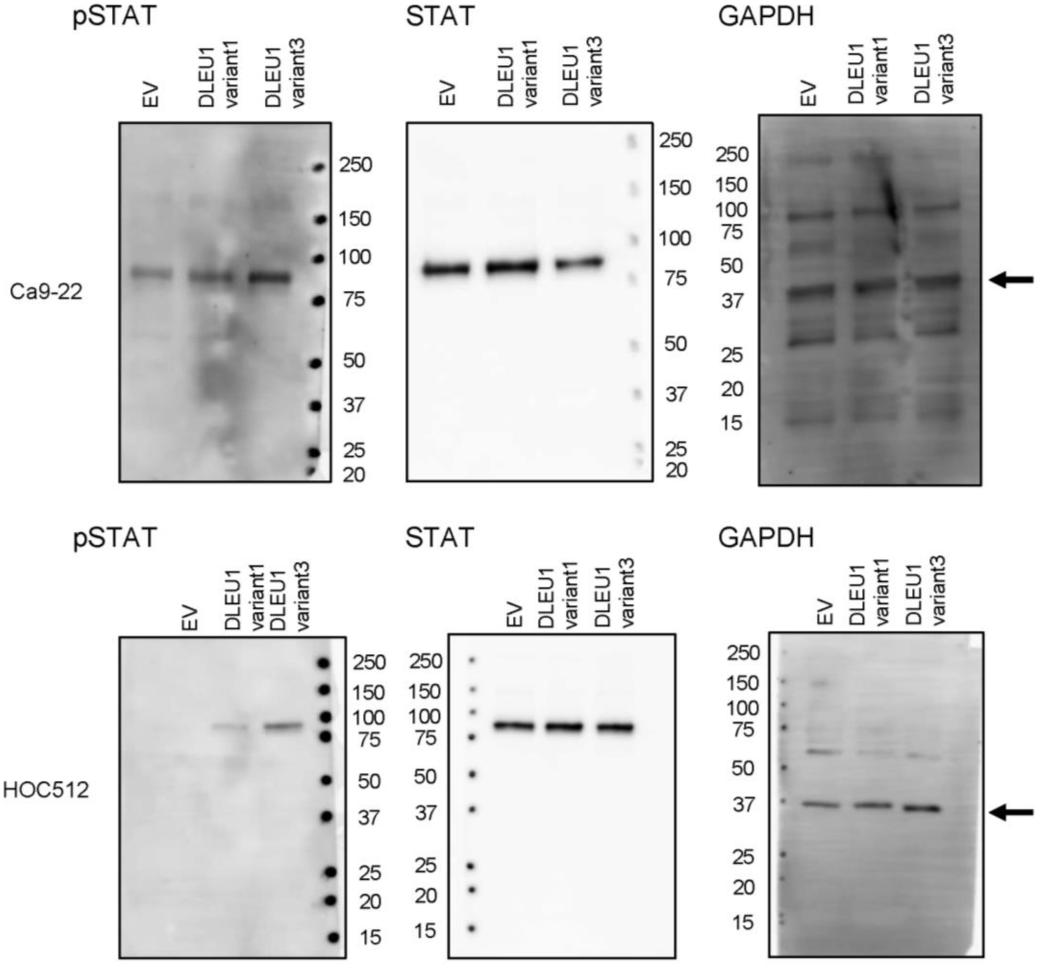

Hatanaka *et al*. Original images of western blots

Figure 6b

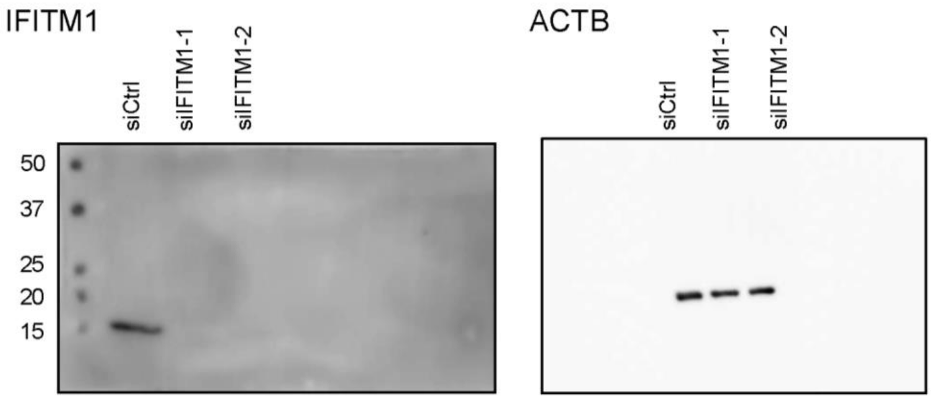

Supplementary Figure S4b

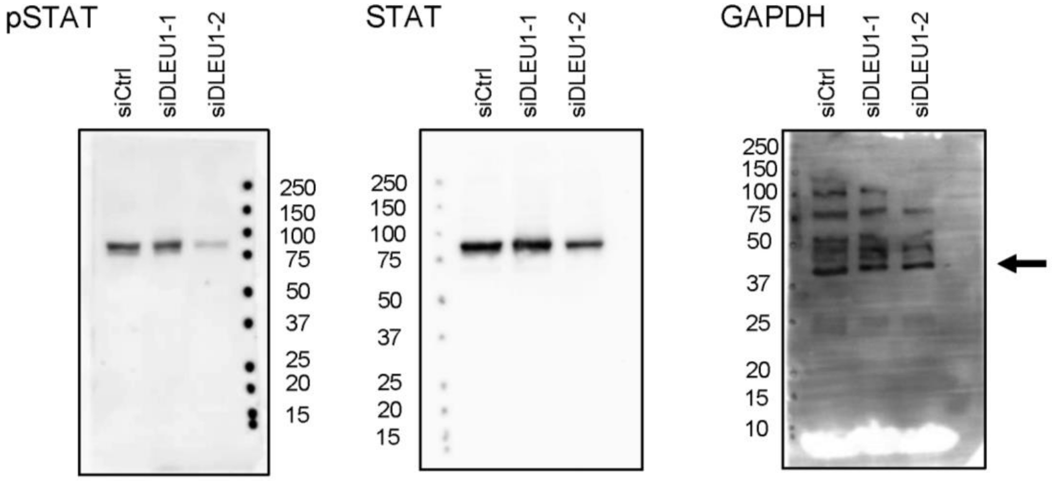

Note: In Figure 4d, we analyzed the effects of ectopic expression of DLEU1 variants 1 (NR_109973) and 3 (NR_109974) and obtained similar results. Because variant 1 was dominantly expressed in OSCC cells, we show results using variant 1 in the main figure.

Supplementary Table S1. Top 300 genes whose H3K27ac levels were decreased by DLEU1 knockdown in HSC3 cells

GeneSymbol	GeneName	PANTHER Gene ID
OSER1	Oxidative stress-responsive serine-rich protein 1;OSER1;ortholog	HUMAN|HGNC=16105|UniProtKB=Q9NX31
ZFP36L1	mRNA decay activator protein ZFP36L1;ZFP36L1;ortholog	HUMAN|HGNC=1107|UniProtKB=Q07352
LY6E	Lymphocyte antigen 6E;LY6E;ortholog	HUMAN|HGNC=6727|UniProtKB=Q16553
SNX12	Sorting nexin-12;SNX12;ortholog	HUMAN|HGNC=14976|UniProtKB=Q9UMY4
SRSF11	Serine/arginine-rich splicing factor 11;SRSF11;ortholog	HUMAN|HGNC=10782|UniProtKB=Q05519
WASL	Neural Wiskott-Aldrich syndrome protein;WASL;ortholog	HUMAN|HGNC=12735|UniProtKB=O00401
PHLDA3	Pleckstrin homology-like domain family A member 3;PHLDA3;ortholog	HUMAN|HGNC=8934|UniProtKB=Q9Y5J5
MYADM	Myeloid-associated differentiation marker;MYADM;ortholog	HUMAN|HGNC=7544|UniProtKB=Q96S97
BICD1	Protein bicaudal D homolog 1;BICD1;ortholog	HUMAN|HGNC=1049|UniProtKB=Q96G01
DHCR24	Delta(24)-sterol reductase;DHCR24;ortholog	HUMAN|HGNC=2859|UniProtKB=Q15392
JMY	Junction-mediating and -regulatory protein;JMY;ortholog	HUMAN|HGNC=28916|UniProtKB=Q8N9B5
SYNE2	Nesprin-2;SYNE2;ortholog	HUMAN|HGNC=17084|UniProtKB=Q8WXH0
ZNF317	Zinc finger protein 317;ZNF317;ortholog	HUMAN|HGNC=13507|UniProtKB=Q96PQ6
ACSS2	Acetyl-coenzyme A synthetase, cytoplasmic;ACSS2;ortholog	HUMAN|HGNC=15814|UniProtKB=Q9NR19
SIX4	Homeobox protein SIX4;SIX4;ortholog	HUMAN|HGNC=10890|UniProtKB=Q9UIU6
LGALS3	Galectin-3;LGALS3;ortholog	HUMAN|HGNC=6563|UniProtKB=P17931
B3GNT5	Lactosylceramide 1,3-N-acetyl-beta-D-glucosaminyltransferase;B3GNT5;ortholog	HUMAN|HGNC=15684|UniProtKB=Q9BYG0
OAS1	2'-5'-oligoadenylate synthase 1;OAS1;ortholog	HUMAN|HGNC=8086|UniProtKB=P00973
POLG	DNA polymerase subunit gamma-1;POLG;ortholog	HUMAN|HGNC=9179|UniProtKB=P54098
DCT	L-dopachrome tautomerase;DCT;ortholog	HUMAN|HGNC=2709|UniProtKB=P40126
SEC61B	Protein transport protein Sec61 subunit beta;SEC61B;ortholog	HUMAN|HGNC=16993|UniProtKB=P60468
ATP6V1E1	V-type proton ATPase subunit E 1;ATP6V1E1;ortholog	HUMAN|HGNC=857|UniProtKB=P36543
SHF	SH2 domain-containing adapter protein F;SHF;ortholog	HUMAN|HGNC=25116|UniProtKB=Q7M4L6
THOC3	THO complex subunit 3;THOC3;ortholog	HUMAN|HGNC=19072|UniProtKB=Q96J01
DCK	Deoxycytidine kinase;DCK;ortholog	HUMAN|HGNC=2704|UniProtKB=P27707
JMJD1C	Probable JmjC domain-containing histone demethylation protein 2C;JMJD1C;ortholog	HUMAN|HGNC=12313|UniProtKB=Q15652
PFN2	Profilin-2;PFN2;ortholog	HUMAN|HGNC=8882|UniProtKB=P35080
LASP1	LIM and SH3 domain protein 1;LASP1;ortholog	HUMAN|HGNC=6513|UniProtKB=Q14847
PTPDC1	Protein tyrosine phosphatase domain-containing protein 1;PTPDC1;ortholog	HUMAN|HGNC=30184|UniProtKB=A2A3K4
TSG101	Tumor susceptibility gene 101 protein;TSG101;ortholog	HUMAN|HGNC=15971|UniProtKB=Q99816

FBXO5	F-box only protein 5;FBXO5;ortholog	HUMAN|HGNC=13584|UniProtKB=Q9UKT4
AJUBA	LIM domain-containing protein ajuba;AJUBA;ortholog	HUMAN|HGNC=20250|UniProtKB=Q96IF1
OASL	2'-5'-oligoadenylate synthase-like protein;OASL;ortholog	HUMAN|HGNC=8090|UniProtKB=Q15646
PARP12	Poly [ADP-ribose] polymerase 12;PARP12;ortholog	HUMAN|HGNC=21919|UniProtKB=Q9H0J9
DOK1	Ecto-ADP-ribosyltransferase 4;ART4;ortholog	HUMAN|HGNC=726|UniProtKB=Q93070
RNF39	RING finger protein 39;RNF39;ortholog	HUMAN|HGNC=18064|UniProtKB=Q9H2S5
HIST1H2AC	Histone H2A type 1-C;HIST1H2AC;ortholog	HUMAN|HGNC=4733|UniProtKB=Q93077
MED21	Mediator of RNA polymerase II transcription subunit 21;MED21;ortholog	HUMAN|HGNC=11473|UniProtKB=Q13503
SMIM26	Small integral membrane protein 26;SMIM26;ortholog	HUMAN|HGNC=43430|UniProtKB=A0A096LP01
IFI27	Interferon alpha-inducible protein 27, mitochondrial;IFI27;ortholog	HUMAN|HGNC=5397|UniProtKB=P40305
GADD45B	Growth arrest and DNA damage-inducible protein GADD45 beta;GADD45B;ortholog	HUMAN|HGNC=4096|UniProtKB=O75293
PPP1R13L	RelA-associated inhibitor;PPP1R13L;ortholog	HUMAN|HGNC=18838|UniProtKB=Q8WUF5
DCAF11	DDB1- and CUL4-associated factor 11;DCAF11;ortholog	HUMAN|HGNC=20258|UniProtKB=Q8TEB1
PRMT5	Protein arginine N-methyltransferase 5;PRMT5;ortholog	HUMAN|HGNC=10894|UniProtKB=O14744
DDX47	Probable ATP-dependent RNA helicase DDX47;DDX47;ortholog	HUMAN|HGNC=18682|UniProtKB=Q9H0S4
SRGAP1	SLIT-ROBO Rho GTPase-activating protein 1;SRGAP1;ortholog	HUMAN|HGNC=17382|UniProtKB=Q7Z6B7
STRAP	Tetratricopeptide repeat protein 5;TTC5;ortholog	HUMAN|HGNC=19274|UniProtKB=Q8N0Z6
VTA1	Vacuolar protein sorting-associated protein VTA1 homolog;VTA1;ortholog	HUMAN|HGNC=20954|UniProtKB=Q9NP79
ACVR1B	Activin receptor type-1B;ACVR1B;ortholog	HUMAN|HGNC=172|UniProtKB=P36896
GLUL	Glutamine synthetase;GLUL;ortholog	HUMAN|HGNC=4341|UniProtKB=P15104
IER5	Immediate early response gene 5 protein;IER5;ortholog	HUMAN|HGNC=5393|UniProtKB=Q5VY09
CEBPD	CCAAT/enhancer-binding protein delta;CEBPD;ortholog	HUMAN|HGNC=1835|UniProtKB=P49716
PRKDC	DNA-dependent protein kinase catalytic subunit;PRKDC;ortholog	HUMAN|HGNC=9413|UniProtKB=P78527
BTN3A1	Butyrophilin subfamily 3 member A1;BTN3A1;ortholog	HUMAN|HGNC=1138|UniProtKB=O00481
KTN1	Kinectin;KTN1;ortholog	HUMAN|HGNC=6467|UniProtKB=Q86UP2
IFIT1	Interferon-induced protein with tetratricopeptide repeats 1;IFIT1;ortholog	HUMAN|HGNC=5407|UniProtKB=P09914
CD109	CD109 antigen;CD109;ortholog	HUMAN|HGNC=21685|UniProtKB=Q6YHK3
PGRMC2	Membrane-associated progesterone receptor component 2;PGRMC2;ortholog	HUMAN|HGNC=16089|UniProtKB=O15173
TDRD7	Tudor domain-containing protein 7;TDRD7;ortholog	HUMAN|HGNC=30831|UniProtKB=Q8NHU6
CCDC50	Coiled-coil domain-containing protein 50;CCDC50;ortholog	HUMAN|HGNC=18111|UniProtKB=Q8IVM0
NDUFS2	NADH dehydrogenase [ubiquinone] iron-sulfur protein 2, mitochondrial;NDUFS2;ortholog	HUMAN|HGNC=7708|UniProtKB=O75306
RPS29	40S ribosomal protein S29;RPS29;ortholog	HUMAN|HGNC=10419|UniProtKB=P62273

FGGY	FGGY carbohydrate kinase domain-containing protein;FGGY;ortholog	HUMAN|HGNC=25610|UniProtKB=Q96C11
TPST2	Protein-tyrosine sulfotransferase 2;TPST2;ortholog	HUMAN|HGNC=12021|UniProtKB=O60704
RAB21	Ras-related protein Rab-21;RAB21;ortholog	HUMAN|HGNC=18263|UniProtKB=Q9UL25
EFNA1	Ephrin-A1;EFNA1;ortholog	HUMAN|HGNC=3221|UniProtKB=P20827
NFKBIA	NF-kappa-B inhibitor alpha;NFKBIA;ortholog	HUMAN|HGNC=7797|UniProtKB=P25963
DBP	D site-binding protein;DBP;ortholog	HUMAN|HGNC=2697|UniProtKB=Q10586
PHF13	PHD finger protein 13;PHF13;ortholog	HUMAN|HGNC=22983|UniProtKB=Q86YI8
ATP9A	Probable phospholipid-transporting ATPase IIA;ATP9A;ortholog	HUMAN|HGNC=13540|UniProtKB=O75110
MX1	Interferon-induced GTP-binding protein Mx1;MX1;ortholog	HUMAN|HGNC=7532|UniProtKB=P20591
PPFIA1	Liprin-alpha-1;PPFIA1;ortholog	HUMAN|HGNC=9245|UniProtKB=Q13136
PHPT1	14 kDa phosphohistidine phosphatase;PHPT1;ortholog	HUMAN|HGNC=30033|UniProtKB=Q9NRX4
MX2	Interferon-induced GTP-binding protein Mx2;MX2;ortholog	HUMAN|HGNC=7533|UniProtKB=P20592
SNRPA1	U2 small nuclear ribonucleoprotein A';SNRPA1;ortholog	HUMAN|HGNC=11152|UniProtKB=P09661
GAS2L1	GAS2-like protein 1;GAS2L1;ortholog	HUMAN|HGNC=16955|UniProtKB=Q99501
IFITM3	Interferon-induced transmembrane protein 3;IFITM3;ortholog	HUMAN|HGNC=5414|UniProtKB=Q01628
MUM1	PWWP domain-containing protein MUM1;MUM1;ortholog	HUMAN|HGNC=29641|UniProtKB=Q2TAK8
SELENOF	Selenoprotein F;SELENOF;ortholog	HUMAN|Gene=SEP15|UniProtKB=O60613
CXCL1	Growth-regulated alpha protein;CXCL1;ortholog	HUMAN|HGNC=4602|UniProtKB=P09341
KLF10	Krueppel-like factor 10;KLF10;ortholog	HUMAN|HGNC=11810|UniProtKB=Q13118
IFI44L	Interferon-induced protein 44-like;IFI44L;ortholog	HUMAN|HGNC=17817|UniProtKB=Q53G44
CALR	Calreticulin;CALR;ortholog	HUMAN|HGNC=1455|UniProtKB=P27797
SLC35B4	UDP-xylose and UDP-N-acetylglucosamine transporter;SLC35B4;ortholog	HUMAN|HGNC=20584|UniProtKB=Q969S0
FLOT1	Folate transporter 1;SLC19A1;ortholog	HUMAN|HGNC=10937|UniProtKB=P41440
GGNBP2	Gametogenetin-binding protein 2;GGNBP2;ortholog	HUMAN|HGNC=19357|UniProtKB=Q9H3C7
ELF3	ETS-related transcription factor Elf-3;ELF3;ortholog	HUMAN|HGNC=3318|UniProtKB=P78545
ZBTB41	Zinc finger and BTB domain-containing protein 41;ZBTB41;ortholog	HUMAN|HGNC=24819|UniProtKB=Q5SVQ8
EIF4A1	Eukaryotic initiation factor 4A-I;EIF4A1;ortholog	HUMAN|HGNC=3282|UniProtKB=P60842
SAP30	Histone deacetylase complex subunit SAP30;SAP30;ortholog	HUMAN|HGNC=10532|UniProtKB=O75446
SERINC1	Serine incorporator 1;SERINC1;ortholog	HUMAN|HGNC=13464|UniProtKB=Q9NRX5
ADAR	Double-stranded RNA-specific adenosine deaminase;ADAR;ortholog	HUMAN|HGNC=225|UniProtKB=P55265
DDX58	Probable ATP-dependent RNA helicase DDX58;DDX58;ortholog	HUMAN|HGNC=19102|UniProtKB=O95786
DHX40	Probable ATP-dependent RNA helicase DHX40;DHX40;ortholog	HUMAN|HGNC=18018|UniProtKB=Q8IX18
EPN3	Epsin-3;EPN3;ortholog	HUMAN|HGNC=18235|UniProtKB=Q9H201

MRPS22	28S ribosomal protein S22, mitochondrial;MRPS22;ortholog	HUMAN|HGNC=14508|UniProtKB=P82650
PSMA3	Proteasome subunit alpha type-3;PSMA3;ortholog	HUMAN|HGNC=9532|UniProtKB=P25788
PIP5K1A	Phosphatidylinositol 4-phosphate 5-kinase type-1 alpha;PIP5K1A;ortholog	HUMAN|HGNC=8994|UniProtKB=Q99755
CAPZA1	F-actin-capping protein subunit alpha-1;CAPZA1;ortholog	HUMAN|HGNC=1488|UniProtKB=P52907
SMARCA2	Probable global transcription activator SNF2L2;SMARCA2;ortholog	HUMAN|HGNC=11098|UniProtKB=P51531
ARHGAP29	Rho GTPase-activating protein 29;ARHGAP29;ortholog	HUMAN|HGNC=30207|UniProtKB=Q52LW3
CHCHD2	Coiled-coil-helix-coiled-coil-helix domain-containing protein 2;CHCHD2;ortholog	HUMAN|HGNC=21645|UniProtKB=Q9Y6H1
ZBTB20	Zinc finger and BTB domain-containing protein 20;ZBTB20;ortholog	HUMAN|HGNC=13503|UniProtKB=Q9HC78
SEC22B	Vesicle-trafficking protein SEC22b;SEC22B;ortholog	HUMAN|HGNC=10700|UniProtKB=O75396
MON1B	Vacuolar fusion protein MON1 homolog B;MON1B;ortholog	HUMAN|HGNC=25020|UniProtKB=Q7L1V2
POLR3E	DNA-directed RNA polymerase III subunit RPC5;POLR3E;ortholog	HUMAN|HGNC=30347|UniProtKB=Q9NVU0
HSPA6	Heat shock 70 kDa protein 6;HSPA6;ortholog	HUMAN|HGNC=5239|UniProtKB=P17066
ANKRD65	Ankyrin repeat domain-containing protein 65;ANKRD65;ortholog	HUMAN|HGNC=42950|UniProtKB=E5RJM6
EEF1A1	Elongation factor 1-alpha 1;EEF1A1;ortholog	HUMAN|HGNC=3189|UniProtKB=P68104
EFCAB11	EF-hand calcium-binding domain-containing protein 11;EFCAB11;ortholog	HUMAN|HGNC=20357|UniProtKB=Q9BUY7
SWAP70	Switch-associated protein 70;SWAP70;ortholog	HUMAN|HGNC=17070|UniProtKB=Q9UH65
PIM1	Serine/threonine-protein kinase pim-1;PIM1;ortholog	HUMAN|HGNC=8986|UniProtKB=P11309
GBP4	Guanylate-binding protein 4;GBP4;ortholog	HUMAN|HGNC=20480|UniProtKB=Q96PP9
CDCA2	Cell division cycle-associated protein 2;CDCA2;ortholog	HUMAN|HGNC=14623|UniProtKB=Q69YH5
TNFAIP8	Tumor necrosis factor alpha-induced protein 8;TNFAIP8;ortholog	HUMAN|HGNC=17260|UniProtKB=O95379
ANG	Angiogenin;ANG;ortholog	HUMAN|HGNC=483|UniProtKB=P03950
TRIP10	Cdc42-interacting protein 4;TRIP10;ortholog	HUMAN|HGNC=12304|UniProtKB=Q15642
IFIT3	Interferon-induced protein with tetratricopeptide repeats 3;IFIT3;ortholog	HUMAN|HGNC=5411|UniProtKB=O14879
VCL	Vinculin;VCL;ortholog	HUMAN|HGNC=12665|UniProtKB=P18206
ATF3	Cyclic AMP-dependent transcription factor ATF-3;ATF3;ortholog	HUMAN|HGNC=785|UniProtKB=P18847
ARAP1	Arf-GAP with Rho-GAP domain, ANK repeat and PH domain-containing protein 1;ARAP1;ortholog	HUMAN|HGNC=16925|UniProtKB=Q96P48
ETV6	Transcription factor ETV6;ETV6;ortholog	HUMAN|HGNC=3495|UniProtKB=P41212
BCL2L13	Bcl-2-like protein 13;BCL2L13;ortholog	HUMAN|HGNC=17164|UniProtKB=Q9BXK5
CDKN2C	Cyclin-dependent kinase 4 inhibitor C;CDKN2C;ortholog	HUMAN|HGNC=1789|UniProtKB=P42773
PYM1	Partner of Y14 and mago;PYM1;ortholog	HUMAN|HGNC=30258|UniProtKB=Q9BRP8
ZCCHC2	Zinc finger CCHC domain-containing protein 2;ZCCHC2;ortholog	HUMAN|HGNC=22916|UniProtKB=Q9C0B9
TARS2	Threonine--tRNA ligase, mitochondrial;TARS2;ortholog	HUMAN|HGNC=30740|UniProtKB=Q9BW92

AHCYL2	Adenosylhomocysteinase 3;AHCYL2;ortholog	HUMAN|HGNC=22204|UniProtKB=Q96HN2
GABARAPL1	Gamma-aminobutyric acid receptor-associated protein-like 1;GABARAPL1;ortholog	HUMAN|HGNC=4068|UniProtKB=Q9H0R8
RMDN2	Regulator of microtubule dynamics protein 2;RMDN2;ortholog	HUMAN|HGNC=26567|UniProtKB=Q96LZ7
PSMD12	26S proteasome non-ATPase regulatory subunit 12;PSMD12;ortholog	HUMAN|HGNC=9557|UniProtKB=O00232
MUM1	Interferon regulatory factor 4;IRF4;ortholog	HUMAN|HGNC=6119|UniProtKB=Q15306
QSER1	Glutamine and serine-rich protein 1;QSER1;ortholog	HUMAN|HGNC=26154|UniProtKB=Q2KHR3
SLC12A7	Solute carrier family 12 member 7;SLC12A7;ortholog	HUMAN|HGNC=10915|UniProtKB=Q9Y666
DHPS	Deoxyhypusine synthase;DHPS;ortholog	HUMAN|HGNC=2869|UniProtKB=P49366
EXOSC8	Exosome complex component RRP43;EXOSC8;ortholog	HUMAN|HGNC=17035|UniProtKB=Q96B26
MYORG	Myogenesis-regulating glycosidase;MYORG;ortholog	HUMAN|HGNC=19918|UniProtKB=Q6NSJ0
HLA-F	HLA class I histocompatibility antigen, alpha chain F;HLA-F;ortholog	HUMAN|HGNC=4963|UniProtKB=P30511
HIBADH	3-hydroxyisobutyrate dehydrogenase, mitochondrial;HIBADH;ortholog	HUMAN|HGNC=4907|UniProtKB=P31937
ZNF391	Zinc finger protein 391;ZNF391;ortholog	HUMAN|HGNC=18779|UniProtKB=Q9UJN7
HNRNPC	Heterogeneous nuclear ribonucleoproteins C1/C2;HNRNPC;ortholog	HUMAN|HGNC=5035|UniProtKB=P07910
ATG14	Beclin 1-associated autophagy-related key regulator;ATG14;ortholog	HUMAN|HGNC=19962|UniProtKB=Q6ZNE5
STRAP	Serine-threonine kinase receptor-associated protein;STRAP;ortholog	HUMAN|HGNC=30796|UniProtKB=Q9Y3F4
XBP1	X-box-binding protein 1;XBP1;ortholog	HUMAN|HGNC=12801|UniProtKB=P17861
NINJ1	Ninjurin-1;NINJ1;ortholog	HUMAN|HGNC=7824|UniProtKB=Q92982
SCARB2	Lysosome membrane protein 2;SCARB2;ortholog	HUMAN|HGNC=1665|UniProtKB=Q14108
SRSF5	Serine/arginine-rich splicing factor 5;SRSF5;ortholog	HUMAN|HGNC=10787|UniProtKB=Q13243
TTL	Tubulin--tyrosine ligase;TTL;ortholog	HUMAN|HGNC=21586|UniProtKB=Q8NG68
SARNP	SAP domain-containing ribonucleoprotein;SARNP;ortholog	HUMAN|HGNC=24432|UniProtKB=P82979
ANKRD12	Ankyrin repeat domain-containing protein 12;ANKRD12;ortholog	HUMAN|HGNC=29135|UniProtKB=Q6UB98
FOXO1	Forkhead box protein O1;FOXO1;ortholog	HUMAN|HGNC=3819|UniProtKB=Q12778
FLOT1	Flotillin-1;FLOT1;ortholog	HUMAN|HGNC=3757|UniProtKB=O75955
ZNF385A	Zinc finger protein 385A;ZNF385A;ortholog	HUMAN|HGNC=17521|UniProtKB=Q96PM9
FBXO32	F-box only protein 32;FBXO32;ortholog	HUMAN|HGNC=16731|UniProtKB=Q969P5
UGCG	Ceramide glucosyltransferase;UGCG;ortholog	HUMAN|HGNC=12524|UniProtKB=Q16739
RTRAF	RNA transcription, translation and transport factor protein;RTRAF;ortholog	HUMAN|HGNC=23169|UniProtKB=Q9Y224
LRRC23	Leucine-rich repeat-containing protein 23;LRRC23;ortholog	HUMAN|HGNC=19138|UniProtKB=Q53EV4
DYNLRB1	Dynein light chain roadblock-type 1;DYNLRB1;ortholog	HUMAN|HGNC=15468|UniProtKB=Q9NP97
F11R	Junctional adhesion molecule A;F11R;ortholog	HUMAN|HGNC=14685|UniProtKB=Q9Y624
PPP1R26	Protein phosphatase 1 regulatory subunit 26;PPP1R26;ortholog	HUMAN|HGNC=29089|UniProtKB=Q5T8A7

PPWD1	Peptidylprolyl isomerase domain and WD repeat-containing protein 1;PPWD1;ortholog	HUMAN|HGNC=28954|UniProtKB=Q96BP3
GJC1	Gap junction gamma-1 protein;GJC1;ortholog	HUMAN|HGNC=4280|UniProtKB=P36383
NBAS	Neuroblastoma-amplified sequence;NBAS;ortholog	HUMAN|HGNC=15625|UniProtKB=A2RRP1
NOXA1	NADPH oxidase activator 1;NOXA1;ortholog	HUMAN|HGNC=10668|UniProtKB=Q86UR1
DBP	Peroxisomal multifunctional enzyme type 2;HSD17B4;ortholog	HUMAN|HGNC=5213|UniProtKB=P51659
OAS2	2'-5'-oligoadenylate synthase 2;OAS2;ortholog	HUMAN|HGNC=8087|UniProtKB=P29728
KLRG1	Killer cell lectin-like receptor subfamily G member 1;KLRG1;ortholog	HUMAN|HGNC=6380|UniProtKB=Q96E93
IFITM1	Interferon-induced transmembrane protein 1;IFITM1;ortholog	HUMAN|HGNC=5412|UniProtKB=P13164
TAF2	Transcription initiation factor TFIID subunit 2;TAF2;ortholog	HUMAN|HGNC=11536|UniProtKB=Q6P1X5
KIF14	Kinesin-like protein KIF14;KIF14;ortholog	HUMAN|HGNC=19181|UniProtKB=Q15058
JAG1	Protein jagged-1;JAG1;ortholog	HUMAN|HGNC=6188|UniProtKB=P78504
MDH1B	Putative malate dehydrogenase 1B;MDH1B;ortholog	HUMAN|HGNC=17836|UniProtKB=Q5I0G3
ZDHHC20	Palmitoyltransferase ZDHHC20;ZDHHC20;ortholog	HUMAN|HGNC=20749|UniProtKB=Q5W0Z9
DDX60	Probable ATP-dependent RNA helicase DDX60;DDX60;ortholog	HUMAN|HGNC=25942|UniProtKB=Q8IY21
LAMTOR1	Ragulator complex protein LAMTOR1;LAMTOR1;ortholog	HUMAN|HGNC=26068|UniProtKB=Q6IAA8
FAM129A	Protein Niban;FAM129A;ortholog	HUMAN|HGNC=16784|UniProtKB=Q9BZQ8
CAPN1	Calpain-1 catalytic subunit;CAPN1;ortholog	HUMAN|HGNC=1476|UniProtKB=P07384
FKBP15	FK506-binding protein 15;FKBP15;ortholog	HUMAN|HGNC=23397|UniProtKB=Q5T1M5
NRDC	Nardilysin;NRDC;ortholog	HUMAN|HGNC=7995|UniProtKB=O43847
CTC1	CST complex subunit CTC1;CTC1;ortholog	HUMAN|HGNC=26169|UniProtKB=Q2NKJ3
ALOXE3	Hydroperoxide isomerase ALOXE3;ALOXE3;ortholog	HUMAN|HGNC=13743|UniProtKB=Q9BYJ1
WEE1	Wee1-like protein kinase;WEE1;ortholog	HUMAN|HGNC=12761|UniProtKB=P30291
GPRC5A	Retinoic acid-induced protein 3;GPRC5A;ortholog	HUMAN|HGNC=9836|UniProtKB=Q8NFJ5
HECTD2	Probable E3 ubiquitin-protein ligase HECTD2;HECTD2;ortholog	HUMAN|HGNC=26736|UniProtKB=Q5U5R9
HIST1H4J	Histone H4;HIST1H4A;ortholog	HUMAN|HGNC=20510|UniProtKB=P62805
TNFRSF1A	Tumor necrosis factor receptor superfamily member 1A;TNFRSF1A;ortholog	HUMAN|HGNC=11916|UniProtKB=P19438
RPL37A	60S ribosomal protein L37a;RPL37A;ortholog	HUMAN|HGNC=10348|UniProtKB=P61513
SLC25A25	Calcium-binding mitochondrial carrier protein SCaMC-2;SLC25A25;ortholog	HUMAN|HGNC=20663|UniProtKB=Q6KCM7
PGK1	Phosphoglycerate kinase 1;PGK1;ortholog	HUMAN|HGNC=8896|UniProtKB=P00558
MRPS35	28S ribosomal protein S28, mitochondrial;MRPS28;ortholog	HUMAN|HGNC=14513|UniProtKB=Q9Y2Q9
ARL4C	ADP-ribosylation factor-like protein 4C;ARL4C;ortholog	HUMAN|HGNC=698|UniProtKB=P56559
TRA2B	Transformer-2 protein homolog beta;TRA2B;ortholog	HUMAN|HGNC=10781|UniProtKB=P62995
ATP11B	Probable phospholipid-transporting ATPase IF;ATP11B;ortholog	HUMAN|HGNC=13553|UniProtKB=Q9Y2G3

FBXW9	F-box/WD repeat-containing protein 9;FBXW9;ortholog	HUMAN|HGNC=28136|UniProtKB=Q5XUX1
KIF20B	Kinesin-like protein KIF20B;KIF20B;ortholog	HUMAN|HGNC=7212|UniProtKB=Q96Q89
ARVCF	Armadillo repeat protein deleted in velo-cardio-facial syndrome;ARVCF;ortholog	HUMAN|HGNC=728|UniProtKB=O00192
RGL2	Ral guanine nucleotide dissociation stimulator-like 2;RGL2;ortholog	HUMAN|HGNC=9769|UniProtKB=O15211
MRPS16	28S ribosomal protein S16, mitochondrial;MRPS16;ortholog	HUMAN|HGNC=14048|UniProtKB=Q9Y3D3
NDUFV1	NADH dehydrogenase [ubiquinone] flavoprotein 1, mitochondrial;NDUFV1;ortholog	HUMAN|HGNC=7716|UniProtKB=P49821
CAPRIN1	Caprin-1;CAPRIN1;ortholog	HUMAN|HGNC=6743|UniProtKB=Q14444
CYP51A1	Lanosterol 14-alpha demethylase;CYP51A1;ortholog	HUMAN|HGNC=2649|UniProtKB=Q16850
DOK1	Docking protein 1;DOK1;ortholog	HUMAN|HGNC=2990|UniProtKB=Q99704
TRIM7	E3 ubiquitin-protein ligase TRIM7;TRIM7;ortholog	HUMAN|HGNC=16278|UniProtKB=Q9C029
AK4	Adenylate kinase 4, mitochondrial;AK4;ortholog	HUMAN|HGNC=363|UniProtKB=P27144
ARID4A	AT-rich interactive domain-containing protein 4A;ARID4A;ortholog	HUMAN|HGNC=9885|UniProtKB=P29374
FAM219B	Protein FAM219B;FAM219B;ortholog	HUMAN|HGNC=24695|UniProtKB=Q5XKK7
RECQL	ATP-dependent DNA helicase Q1;RECQL;ortholog	HUMAN|HGNC=9948|UniProtKB=P46063
RIC1	RAB6A-GEF complex partner protein 1;RIC1;ortholog	HUMAN|HGNC=17686|UniProtKB=Q4ADV7
PLAU	Urokinase-type plasminogen activator;PLAU;ortholog	HUMAN|HGNC=9052|UniProtKB=P00749
SEMA6D	Semaphorin-6D;SEMA6D;ortholog	HUMAN|HGNC=16770|UniProtKB=Q8NFY4
NFKB2	Nuclear factor NF-kappa-B p100 subunit;NFKB2;ortholog	HUMAN|HGNC=7795|UniProtKB=Q00653
EDAR	Tumor necrosis factor receptor superfamily member EDAR;EDAR;ortholog	HUMAN|HGNC=2895|UniProtKB=Q9UNE0
ATP5F1B	ATP synthase subunit beta, mitochondrial;ATP5F1B;ortholog	HUMAN|HGNC=830|UniProtKB=P06576
LYSMD3	LysM and putative peptidoglycan-binding domain-containing protein 3;LYSMD3;ortholog	HUMAN|HGNC=26969|UniProtKB=Q7Z3D4
GJC1	Gap junction delta-3 protein;GJD3;ortholog	HUMAN|HGNC=19147|UniProtKB=Q8N144
AEBP2	Zinc finger protein AEBP2;AEBP2;ortholog	HUMAN|HGNC=24051|UniProtKB=Q6ZN18
ARMCX3	Armadillo repeat-containing X-linked protein 3;ARMCX3;ortholog	HUMAN|HGNC=24065|UniProtKB=Q9UH62
NFE2L1	Endoplasmic reticulum membrane sensor NFE2L1;NFE2L1;ortholog	HUMAN|HGNC=7781|UniProtKB=Q14494
HMGN4	High mobility group nucleosome-binding domain-containing protein 4;HMGN4;ortholog	HUMAN|HGNC=4989|UniProtKB=O00479
CRYBG1	Beta/gamma crystallin domain-containing protein 1;CRYBG1;ortholog	HUMAN|HGNC=356|UniProtKB=Q9Y4K1
PSMB5	Proteasome subunit beta type-5;PSMB5;ortholog	HUMAN|HGNC=9542|UniProtKB=P28074
IFI44	Interferon-induced protein 44;IFI44;ortholog	HUMAN|HGNC=16938|UniProtKB=Q8TCB0
MRPL18	39S ribosomal protein L18, mitochondrial;MRPL18;ortholog	HUMAN|HGNC=14477|UniProtKB=Q9H0U6
PLXDC1	Plexin domain-containing protein 1;PLXDC1;ortholog	HUMAN|HGNC=20945|UniProtKB=Q8IUK5
KIAA0040	Uncharacterized protein KIAA0040;KIAA0040;ortholog	HUMAN|HGNC=28950|UniProtKB=Q15053
PLSCR1	Phospholipid scramblase 1;PLSCR1;ortholog	HUMAN|HGNC=9092|UniProtKB=O15162

PFKFB3	6-phosphofructo-2-kinase/fructose-2,6-bisphosphatase 3;PFKFB3;ortholog	HUMAN|HGNC=8874|UniProtKB=Q16875
GEMIN8	Gem-associated protein 8;GEMIN8;ortholog	HUMAN|HGNC=26044|UniProtKB=Q9NWZ8
LTBP2	Latent-transforming growth factor beta-binding protein 2;LTBP2;ortholog	HUMAN|HGNC=6715|UniProtKB=Q14767
CDC42EP4	Cdc42 effector protein 4;CDC42EP4;ortholog	HUMAN|HGNC=17147|UniProtKB=Q9H3Q1
TMEM191C	Transmembrane protein 191C;TMEM191C;ortholog	HUMAN|HGNC=33601|UniProtKB=A6NGB0
TMEM161B	Transmembrane protein 161B;TMEM161B;ortholog	HUMAN|HGNC=28483|UniProtKB=Q8NDZ6
FAM177A1	Protein FAM177A1;FAM177A1;ortholog	HUMAN|HGNC=19829|UniProtKB=Q8N128
ILF2	Interleukin enhancer-binding factor 2;ILF2;ortholog	HUMAN|HGNC=6037|UniProtKB=Q12905
IRF9	Interferon regulatory factor 9;IRF9;ortholog	HUMAN|HGNC=6131|UniProtKB=Q00978
DROSHA	Ribonuclease 3;DROSHA;ortholog	HUMAN|HGNC=17904|UniProtKB=Q9NRR4
B4GAT1	Beta-1,4-glucuronyltransferase 1;B4GAT1;ortholog	HUMAN|HGNC=15685|UniProtKB=O43505
E2F8	Transcription factor E2F8;E2F8;ortholog	HUMAN|HGNC=24727|UniProtKB=A0AVK6
USPL1	SUMO-specific isopeptidase USPL1;USPL1;ortholog	HUMAN|HGNC=20294|UniProtKB=Q5W0Q7
HMGB2	High mobility group protein B2;HMGB2;ortholog	HUMAN|HGNC=5000|UniProtKB=P26583
MTUS1	Microtubule-associated tumor suppressor 1;MTUS1;ortholog	HUMAN|HGNC=29789|UniProtKB=Q9ULD2
KCTD2	BTB/POZ domain-containing protein KCTD2;KCTD2;ortholog	HUMAN|HGNC=21294|UniProtKB=Q14681
MRPS18A	39S ribosomal protein S18a, mitochondrial;MRPS18A;ortholog	HUMAN|HGNC=14515|UniProtKB=Q9NVS2
PRPF38A	Pre-mRNA-splicing factor 38A;PRPF38A;ortholog	HUMAN|HGNC=25930|UniProtKB=Q8NAV1
CMPK2	UMP-CMP kinase 2, mitochondrial;CMPK2;ortholog	HUMAN|HGNC=27015|UniProtKB=Q5EBM0
STOM	Erythrocyte band 7 integral membrane protein;STOM;ortholog	HUMAN|HGNC=3383|UniProtKB=P27105
RNF44	RING finger protein 44;RNF44;ortholog	HUMAN|HGNC=19180|UniProtKB=Q7L0R7
ADPRM	Manganese-dependent ADP-ribose/CDP-alcohol diphosphatase;ADPRM;ortholog	HUMAN|HGNC=30925|UniProtKB=Q3LIE5
DBP	Vitamin D-binding protein;GC;ortholog	HUMAN|HGNC=4187|UniProtKB=P02774
SF3B1	Splicing factor 3B subunit 1;SF3B1;ortholog	HUMAN|HGNC=10768|UniProtKB=O75533
MECP2	Methyl-CpG-binding protein 2;MECP2;ortholog	HUMAN|HGNC=6990|UniProtKB=P51608
TRA2A	Transformer-2 protein homolog alpha;TRA2A;ortholog	HUMAN|HGNC=16645|UniProtKB=Q13595
HAUS6	HAUS augmin-like complex subunit 6;HAUS6;ortholog	HUMAN|HGNC=25948|UniProtKB=Q7Z4H7
PLEKHH3	Pleckstrin homology domain-containing family H member 3;PLEKHH3;ortholog	HUMAN|HGNC=26105|UniProtKB=Q7Z736
ZNF235	Zinc finger protein 235;ZNF235;ortholog	HUMAN|HGNC=12866|UniProtKB=Q14590
INTS4	Integrator complex subunit 4;INTS4;ortholog	HUMAN|HGNC=25048|UniProtKB=Q96HW7
FAM110C	Protein FAM110C;FAM110C;ortholog	HUMAN|HGNC=33340|UniProtKB=Q1W6H9
NGFR	Tumor necrosis factor receptor superfamily member 16;NGFR;ortholog	HUMAN|HGNC=7809|UniProtKB=P08138
AGAP11	Arf-GAP with GTPase, ANK repeat and PH domain-containing protein 11;AGAP11;ortholog	HUMAN|HGNC=29421|UniProtKB=Q8TF27

TGM2	Protein-glutamine gamma-glutamyltransferase 2;TGM2;ortholog	HUMAN|HGNC=11778|UniProtKB=P21980
TXNRD1	Thioredoxin reductase 1, cytoplasmic;TXNRD1;ortholog	HUMAN|HGNC=12437|UniProtKB=Q16881
OSGEP	Probable tRNA N6-adenosine threonylcarbamoyltransferase;OSGEP;ortholog	HUMAN|HGNC=18028|UniProtKB=Q9NPF4
KLK10	Kallikrein-10;KLK10;ortholog	HUMAN|HGNC=6358|UniProtKB=O43240
EIPR1	EARP-interacting protein;EIPR1;ortholog	HUMAN|HGNC=12383|UniProtKB=Q53HC9
OCEL1	Occludin/ELL domain-containing protein 1;OCEL1;ortholog	HUMAN|HGNC=26221|UniProtKB=Q9H607
VPS8	Vacuolar protein sorting-associated protein 8 homolog;VPS8;ortholog	HUMAN|HGNC=29122|UniProtKB=Q8N3P4
UQCRFS1	Cytochrome b-c1 complex subunit Rieske, mitochondrial;UQCRFS1;ortholog	HUMAN|HGNC=12587|UniProtKB=P47985
FBXO9	F-box only protein 9;FBXO9;ortholog	HUMAN|HGNC=13588|UniProtKB=Q9UK97
MT2A	Metallothionein-2;MT2A;ortholog	HUMAN|HGNC=7406|UniProtKB=P02795
HIST1H2BD	Histone H2B type 1-D;HIST1H2BD;ortholog	HUMAN|HGNC=4747|UniProtKB=P58876
ERCC5	DNA repair protein complementing XP-G cells;ERCC5;ortholog	HUMAN|HGNC=3437|UniProtKB=P28715
ZNF687	Zinc finger protein 687;ZNF687;ortholog	HUMAN|HGNC=29277|UniProtKB=Q8N1G0
CMPK1	UMP-CMP kinase;CMPK1;ortholog	HUMAN|HGNC=18170|UniProtKB=P30085
CSTF1	Cleavage stimulation factor subunit 1;CSTF1;ortholog	HUMAN|HGNC=2483|UniProtKB=Q05048
HIST1H1E	Histone H1.4;HIST1H1E;ortholog	HUMAN|HGNC=4718|UniProtKB=P10412
CLMN	Calmin;CLMN;ortholog	HUMAN|HGNC=19972|UniProtKB=Q96JQ2
C2orf15	Uncharacterized protein C2orf15;C2orf15;ortholog	HUMAN|HGNC=28436|UniProtKB=Q8WU43
KNL1	Kinetochore scaffold 1;KNL1;ortholog	HUMAN|HGNC=24054|UniProtKB=Q8NG31
XAF1	XIAP-associated factor 1;XAF1;ortholog	HUMAN|HGNC=30932|UniProtKB=Q6GPH4
ZNF440	Zinc finger protein 440;ZNF440;ortholog	HUMAN|HGNC=20874|UniProtKB=Q8IYI8
UBE2C	Ubiquitin-conjugating enzyme E2 C;UBE2C;ortholog	HUMAN|HGNC=15937|UniProtKB=O00762
RARS	Arginine--tRNA ligase, cytoplasmic;RARS;ortholog	HUMAN|HGNC=9870|UniProtKB=P54136
PARP14	Poly [ADP-ribose] polymerase 14;PARP14;ortholog	HUMAN|HGNC=29232|UniProtKB=Q460N5
SLC38A2	Sodium-coupled neutral amino acid transporter 2;SLC38A2;ortholog	HUMAN|HGNC=13448|UniProtKB=Q96QD8
KLHL24	Kelch-like protein 24;KLHL24;ortholog	HUMAN|HGNC=25947|UniProtKB=Q6TFL4
CHURC1	Protein Churchill;CHURC1;ortholog	HUMAN|HGNC=20099|UniProtKB=Q8WUH1
NDUFB6	NADH dehydrogenase [ubiquinone] 1 beta subcomplex subunit 6;NDUFB6;ortholog	HUMAN|HGNC=7701|UniProtKB=O95139
SOCS2	Suppressor of cytokine signaling 2;SOCS2;ortholog	HUMAN|HGNC=19382|UniProtKB=O14508
IFI6	Interferon alpha-inducible protein 6;IFI6;ortholog	HUMAN|HGNC=4054|UniProtKB=P09912
TOMM40	Mitochondrial import receptor subunit TOM40 homolog;TOMM40;ortholog	HUMAN|HGNC=18001|UniProtKB=O96008
HELZ2	Helicase with zinc finger domain 2;HELZ2;ortholog	HUMAN|HGNC=30021|UniProtKB=Q9BYK8
APOOL	MICOS complex subunit MIC27;APOOL;ortholog	HUMAN|HGNC=24009|UniProtKB=Q6UXV4

UNC93B1	Protein unc-93 homolog B1;UNC93B1;ortholog	HUMAN|HGNC=13481|UniProtKB=Q9H1C4
MRPS35	28S ribosomal protein S35, mitochondrial;MRPS35;ortholog	HUMAN|HGNC=16635|UniProtKB=P82673
ZNHIT6	Box C/D snoRNA protein 1;ZNHIT6;ortholog	HUMAN|HGNC=26089|UniProtKB=Q9NWK9
TMED7	Transmembrane emp24 domain-containing protein 7;TMED7;ortholog	HUMAN|HGNC=24253|UniProtKB=Q9Y3B3
AGO1	Protein argonaute-1;AGO1;ortholog	HUMAN|HGNC=3262|UniProtKB=Q9UL18
B3GALT4	Beta-1,3-galactosyltransferase 4;B3GALT4;ortholog	HUMAN|HGNC=919|UniProtKB=O96024
SEL1L	Protein sel-1 homolog 1;SEL1L;ortholog	HUMAN|HGNC=10717|UniProtKB=Q9UBV2
FGF19	Fibroblast growth factor 19;FGF19;ortholog	HUMAN|HGNC=3675|UniProtKB=O95750

Supplementary Table S2. Sequences of the primers used in this study

qRT-PCR		
DLEU1	Forward	5'-TCAGAATGCCGACTCTATGCT-3'
	Reverse	5'-GGTGAGGACAGAGTTAAACGC-3'
	Product size	103 bp
OAS1 iso1	Forward	5'-TGTGTGTCCAAGGTGGTAAAG-3'
	Reverse	5'-TGAGAGGACTGAGGAAGACAA-3'
	Product size	97 bp
IFITM3	Forward	5'-TCTTCCAGGCCTATGGATAGAT-3'
	Reverse	5'-GAAGTTGGAGTACGTGGGATAC-3'
	Product size	84 bp
IFITM1	Forward	5'-TGCACAAGGAGGAACATGAG-3'
	Reverse	5'-GTCTCGCTGTGGATGTTGAT-3'
	Product size	91 bp
IFI6	Forward	5'-GCAGCGTCGTCATAGGTAAT-3'
	Reverse	5'-ACTCCTCATCCTCCTCACTATC-3'
	Product size	81 bp
IFIT1 iso1	Forward	5'-CCAACAGTGTAGTAGCCTCAAA-3'
	Reverse	5'-GGTGCGTCCTTAGAAGAAAGAG-3'
	Product size	97 bp
IFNAR1	Forward	5'-CACAGTGGCTCATGCCTATAA-3'
	Reverse	5'-TACCCAGGTTGGTTGATCTTG-3'
	Product size	94 bp
IFMGR2	Forward	5'-CTGGGACTCTGTGTCCATTATC-3'
	Reverse	5'-CTCCGATGGCTTGATCTCTTC-3'
	Product size	121 bp
B2M	Forward	5'-ACTCTCTCTTTCTGGCCTGGA-3'
	Reverse	5'-TCTCTGCTGGATGACGTGAG-3'

	Product size	3883bp
ChIP-qPCR		
IFI6	Forward	5'-ACTAAAAGGGACCCAGGAGTG-3'
	Reverse	5'-ATGAAGTGTGGGCATCAAGC-3'
	Product size	121 bp
IFIT1	Forward	5'-TGTGTGCATGCATGTGTGTG-3'
	Reverse	5'-TTTTCCCATTTCCCTCCCCTTC-3'
	Product size	129 bp
IFITM1	Forward	5'-TGTGTGTGTGTGTGGCTTTG-3'
	Reverse	5'-ACTGGGCTATAGGGAGAATTGC-3'
	Product size	131 bp
OAS1	Forward	5'-CCGCATGCAAATCAACCATG-3'
	Reverse	5'-TGGACTCACCTTTACCACCTTG-3'
	Product size	112 bp

Supplementary Table S3. Sequences of the siRNAs used in this study

siRNA name		
siDLEU1-1	Sense	5'-GAGGGGUCGUCUUAUAGCUAC-3'
	Antisense	5'-AGCUAUAAGACGACCCCUCGG-3'
siDLEU1-2	Sense	5'-CAGGGGUUAUCGCACUUAAGC-3'
	Antisense	5'-UUAAGUGCGAUAACCCCUGUA-3'
siIFITM1-1	Sense	5'-AGGUCCACCGUGAUCAAC-3'
	Antisense	5'-UGUGGAUGUUGAUCACG-3'
siIFITM1-2	Sense	5'-GUCCUGCCCCUAGAUACA-3'
	Antisense	5'-AAACUGCUGUAUCUAGGG-3'
